# Importance of the description of light interception in crop growth models

**DOI:** 10.1093/plphys/kiab113

**Published:** 2021-03-12

**Authors:** Shouyang Liu, Frédéric Baret, Mariem Abichou, Loïc Manceau, Bruno Andrieu, Marie Weiss, Pierre Martre

**Affiliations:** 1 LEPSE, Univ Montpellier, INRAE, Institut Agro Montpellier, Montpellier, France; 2 CAPTE-EMMAH, Université d'Avignon et des Pays de Vaucluse, INRAE, Avignon, France; 3 PheniX, Plant Phenomics Research Centre, Nanjing Agricultural University, Nanjing, China; 4 EcoSys, INRAE, AgroParisTech, Thiverval-Grignon, France

## Abstract

Canopy light interception determines the amount of energy captured by a crop, and is thus critical to modeling crop growth and yield, and may substantially contribute to the prediction uncertainty of crop growth models (CGMs). We thus analyzed the canopy light interception models of the 26 wheat (*Triticum aestivum*) CGMs used by the Agricultural Model Intercomparison and Improvement Project (AgMIP). Twenty-one CGMs assume that the light extinction coefficient (*K*) is constant, varying from 0.37 to 0.80 depending on the model. The other models take into account the illumination conditions and assume either that all green surfaces in the canopy have the same inclination angle (θ) or that θ distribution follows a spherical distribution. These assumptions have not yet been evaluated due to a lack of experimental data. Therefore, we conducted a field experiment with five cultivars with contrasting leaf stature sown at normal and double row spacing, and analyzed θ distribution in the canopies from three-dimensional canopy reconstructions. In all the canopies, θ distribution was well represented by an ellipsoidal distribution. We thus carried out an intercomparison between the light interception models of the AgMIP–Wheat CGMs ensemble and a physically based *K* model with ellipsoidal leaf angle distribution and canopy clumping ($K_{\mathrm{ell}}^{C}$). Results showed that the $K_{\mathrm{ell}}^{C}$ model outperformed current approaches under most illumination conditions and that the uncertainty in simulated wheat growth and final grain yield due to light models could be as high as 45%. Therefore, our results call for an overhaul of light interception models in CGMs.

## Teaser

Describing wheat canopy structure with an ellipsoidal leaf angle distribution improves the prediction accuracy of light interception by wheat canopies.

## Introduction

Crop growth models (CGMs) are popular tools used to optimize crop management, assess the impact of climate change on crop production (Liu et al., 2016), or assist plant breeders (e.g. Martre et al., 2015a, 2015b, 2015c; Chenu et al., 2017). To meet this need, CGMs should be capable of predicting the response of genotypes to various environments (Yin et al., 2003; Parent and Tardieu, 2014; Messina et al., 2018). This requires improved CGMs to reconcile biological realism and parsimony in the description of eco-physiological processes (Hammer et al., 2019) while considering feedbacks between key processes (Tardieu and Parent, 2017). Model parameters should also have sound biological meaning and if possible, be measurable using modern high-through phenotyping methods (Tardieu et al., 2017), be less dependent on the environmental conditions, and allow for more genetic dependency (Hammer et al., 2006, 2016).

This means that CGMs should predict complex traits such as yield with fewer compensations for errors (Muller and Martre, 2019). Among the several processes that need to be described in CGMs, canopy light absorption is very important since it drives the energy available for photosynthesis and transpiration. In the many CGMs that use the classical light use efficiency (LUE) approach to model biomass production (Monteith, 1977), errors in light absorption can easily be compensated for by changes in the LUE or the green area index (GAI). Canopy light absorption in most CGMs is described using the simple big-leaf approach originally proposed by Monsi and Saeki (2005). However, this approach may be too simplistic to accurately account for the impact of illumination conditions (fraction of diffuse incoming radiation and sun position) and canopy structure (primarily GAI, surface inclination angle, and planting arrangement) on light absorption by the canopy (Pury and Farquhar, 1997).

Several alternative approaches to the big-leaf approach have been developed to model canopy light absorption. The most popular ones include the single-layered (Pury and Farquhar, 1997) and multi-layered (Sellers et al., 1992; Wang and Leuning, 1998) sun/shade approach, the three-dimensional (3D) voxel-based canopy description (Sinoquet et al., 2001), and the 3D canopy architecture description (Chelle and Andrieu, 1998). They differ mainly in the level of details with which canopy architecture is described and the approximations in the radiative transfer. The photosynthetic pigments absorb the incoming radiation in the 400–700 nm spectral domain called the photosynthetically active radiation (PAR; McCree, 1972). The fraction of incoming PAR absorbed by the photosynthetically active elements of canopies (FAPAR) quantifies the PAR absorption efficiency (Monteith, 1977; Sinclair and Muchow, 1999). FAPAR results from complex interactions between the incident radiation, characterized by its spectral and its directional distribution, and the structure and biochemical composition of canopies (Hanan and Begue, 1995; Weiss and Baret, 2011).

Canopy structure plays a key role in canopy light interception, whereas there is substantial structural difference among crops. Our focus in this study was on wheat (*Triticum aestivum*), the most surface-grown crop in the world and which contributes ∼20% of the total calories and protein in the human diet (Shiferaw et al., 2013). Because of the strong absorption of PAR radiation by the photosynthetic pigments, multiple scattering contributes only marginally to the radiative transfer, and FAPAR can be closely approximated by the fraction of PAR intercepted by the photosynthetically active radiation (FIPAR) elements of canopies. This approximation is well verified for wheat that does not present glossy or hairy leaves (Andrieu and Baret, 1993; Mõttus et al., 2013).

The Monsi and Saeki (2005) model assume that all elements in the canopy are the same and are randomly distributed in the canopy volume. In these conditions, the incremental change of transmitted PAR, dPAR, due to an incremental increase of canopy leaf area, dL, is proportional to an extinction coefficient K: dPAR=-KdL. The fraction of PAR transmitted to the ground level (τ) is computed by integrating the previous differential equation over canopy depth: $\tau =e^{-K\times L}$. L corresponds to the GAI, which describes the area of the green elements per unit horizontal ground area. Therefore, according to the Monsi and Saeki (2005) model, FIPAR is given by:

(1) $$\mathrm{FIPAR}=\;1-\;\tau =1-e^{-K\times \mathrm{GAI}}$$

A projection function (G) is introduced to account for the actual effective cross section of the elements, which depends on the orientation of the green elements approximated as small flat surfaces and on the direction of the incident light $W\;=\;\left(\beta ,\varphi \right),$ with β and φ being the sun elevation and azimuth angles, respectively (Campbell and Norman, 2012). G is the projected area of a unit GAI onto a surface perpendicular to the incident radiation direction. K is therefore given by:

(2) $$K=C(W)\frac{G(W)}{\sin\beta }$$

where sin⁡β accounts for the optical path that depends on the direction of the incident light and C(W) is a clumping factor that describes the spatial dependency of the positions of the leaves in the canopy (Weiss et al., 2004). It depends on the incident angle W (Andrieu and Sinoquet, 1993). Clumped leaves with $C\left(W\right)\;<1\;$tend to overlap in the W direction, while regularly distributed leaves with $C\left(W\right)\;>\;1\;$tend to avoid themselves. However, for the sake of simplicity, $C\left(W\right)$ is frequently assumed to be independent of direction W. Besides, C(W) depends both on the plant structure, that is, the location of foliage along the plant stems, and on the canopy structure, that is, the distribution of the plants within the canopy (Weiss et al., 2004).

Therefore, modeling light interception requires knowing the distribution of leaf orientation and leaf clumping. Further, the directionality of the incident radiation that governs the interception efficiency at the instantaneous time scale should also be described, including its variation during the day and the growing season. Most CGMs do not account for these additional factors and a thorough quantitative evaluation of the errors of FIPAR estimation in CGMs is still lacking. Therefore, the objective of this study was to analyze the main approaches used in CGMs for computing FIPAR for wheat crops. More than 40 CGMs (White et al., 2011) have been proposed to describe wheat productivity and yield as a function of the environmental conditions. We first reviewed the values of light extinction coefficient reported for wheat and the FIPAR models used in wheat CGMs that are considered in the Agricultural Model Intercomparison and Improvement Project (AgMIP) (Rosenzweig et al., 2013). We then investigated the actual distribution of the inclination of wheat elements (leaves, stems, and ears) and generated a reference dataset using the 3D ADEL–Wheat architectural model (Fournier et al., 2003). We evaluated the accuracy of the FIPAR models in the AgMIP–wheat CGM ensemble at five locations spanning the range of latitudes at which wheat is grown globally. Finally, we discussed our results by highlighting the main factors that drive wheat canopy PAR interception efficiency, with emphasis on the time scale.

### Review of light extinction coefficient values reported for wheat

The approach usually used to estimate *K* is to invert Equation 1 using concurrent measurements of FIPAR and GAI. In most studies, several replicates of FIPAR and GAI measured at several dates along the growth cycle, or across modalities, are used to get robust estimates at the expense of a more limited capacity to describe the possible changes of *K* with time or modalities (Table 1). Alternatively, measuring the vertical profile of FIPAR and GAI was used to invert Equation 1 (Moreau et al., 2012). However, in this case, the possible variation of leaf orientation and clumping with canopy depth may also confound the estimated value of *K*.

**Table 1:** Summary of extinction coefficient K reported for wheat

GLAI, GAI or LAI	Clear Sky	Hour (solar)	Latitude (°)	Longitude (°)	Factors	Growth Stages	Light Extinction Coefficient (unitless)	Reference
NS	NS	11–13	39	−96	2 cultivars 3 densities	From booting to soft dough	0.65–0.89	Asrar et al. (1984)
LAI	Yes	NS	39	−96	2 cultivars 3 densities	From emergence to maturity	0.33–0.84	Fuchs et al. (1984)
LAI	NS	8–16	53	−1	5 cultivars	Before anthesis	0.28–0.44	Green (1989)
LAI	NS	12	52	5	2 nitrogen 2 densities	From emergence to maturity	0.30–0.90	Dreccer et al. (2000)
LAI	NS	11–13	−34	−58	2 nitrogen 2 disease	Heading to anthesis	0.56–0.68	Carretero et al. (2010)
LAI	Yes	11–13	−34	−58	Tall, semi-dwarf, and dwarf isogenic lines	From 30 days after sowing to the time when the canopy reached the maximum LAI	0.48–0.78	Miralles and Slafer (1997)
GLAI	Yes	12	−31	−58	7 cultivars	From emergence to anthesis	0.37–0.46	Calderini et al. (1997)
GLAI	NS	11–13	60	23	3 cultivars 2 nitrogen	From leaf 3 to maturity	0.62–0.79	Muurinen and Peltonen-Sainio (2006)
GAI	NS	10–14	52	0	Growth stage	Between leaf 3 and heading Between heading and anthesis During grain filling	0.46 0.61 0.56	Thorne et al. (1988)
GAI	NS	12	−32	116	3 cultivars	Before heading After heading	0.31–0.60 0.60–0.77	Yunusa et al. (1993)
GAI	NS	12	−35	141	2 years	From emergence to maturity	0.82	O'Connell et al. (2004)
GAI	NS	NS	53	−1	8 cultivars	Beginning of stem extension Anthesis	0.48–0.58 0.53–0.63	Shearman et al. (2005)
GAI	NS	10–14	45–52	3 to −1	16 cultivars 2 nitrogen	Anthesis	0.33–0.54	Moreau et al. (2012)

NS, not specified.

GAI is used in Equation 1 since it accounts for all the green area of leaves, stems, and ears. When *K* is estimated using green leaf area index (GLAI; Calderini et al., 1997; Muurinen and Peltonen-Sainio, 2006) or LAI (Green, 1989; Dreccer, 2000; Carretero et al., 2010), the green stem or ear contribution is not explicitly accounted for. Further, in the case of LAI, the senescent leaves are taken into account although they have no potentials to transform the intercepted radiation into assimilates. Therefore, the use of GLAI or LAI in place of GAI may cause biased estimates of K. However, Table 1 does not show apparent bias in the reported *K* values, probably because the other factors of variability mask this expected effect.

The reported values of K vary between 0.28 and 0.90 with a median value of 0.59 (Table 1). For a given cultivar, K was reported to change between cultivation practices (Asrar et al., 1984; Dreccer, 2000) or with growth stages (Yunusa et al., 1993). Other studies show substantial genotypic differences (Green, 1989; Miralles and Slafer, 1997; Shearman et al., 2005) and strong genotype by environment effect was found in some cases (Moreau et al., 2012) while no substantial cultivar effect was observed in other studies (Yunusa et al., 1993; Calderini et al., 1997; Shearman et al., 2005).

### Review of light interception models in wheat CGMs

We reviewed the canopy light interception models in 26 of the 28 different wheat CGMs used in the AgMIP (Table 2) (Asseng et al., 2013, 2015; Martre et al., 2015a, 2015b, 2015c; Asseng et al., 2019). Two CGMs were not considered (AQUACROP and OLEARY) because they calculate biomass accumulation using a transpiration efficiency approach and thus PAR interception is not calculated *per se* in these CGMs. The AgMIP–Wheat multi-model ensemble comprises >80% of the wheat CGMs currently used (Asseng et al., 2019). We considered FIPAR rather than FAPAR to focus on the way models describe canopy architecture and account for the illumination conditions while not considering the impact of leaf and soil optical properties as required for FAPAR computation. Except for those with a very bright background (white sandy soil or snow), most arable soils have relatively low reflectance values in the PAR spectral domain (Jacquemoud et al., 1992). Further, the green elements strongly absorb light in the PAR domain, resulting in very low reflectance and transmittance values (Jacquemoud and Baret, 1990). In most cases, FIPAR is therefore a close approximation of FAPAR (Bégué et al., 1996; Widlowski, 2010). For the models that describe FAPAR, we, therefore, assumed that the leaves absorb all the radiation (i.e. reflectance and transmittance equal zero) and the soil background is black. This assumption is supported by a sensitivity analysis of the differences between FIPAR and FAPAR (Jacquemoud et al., 2009) in soil reflectance, leaf chlorophyll concentration, GAI, average surface inclination angle, fraction of diffuse radiation, and sun elevation angle (see Supplemental Methods S1; Supplemental Results S1; Supplemental Table S1; Supplemental Figures S1 and S2).

**Table 2:** Summary of the light extinction coefficient, light interception models, and biomass production models of the wheat CGMs of the AgMIP–Wheat multi-model ensemble

Models		Extinction Coefficient			Element Area		FIPAR	FAPAR	Clumping	Biomass Production		Time Step			Reference
Type	Name	Total	Direct	Diffuse	Sheath	Spike				Light Utilization^a^	Light interception^b^	Daily	Hourly	PGI^c^	
Constant	APSIM-Wheat (v7.5)	0.50	–	–	–	–	Yes	–	–	RUE/TE	BLC	Yes	–	–	Meinke et al. (1998)
	APSIM-Wheat 2018	0.50–0.60^d^	–	–	–	–	Yes	–	–	RUE	BLC	Yes	–	–	Brown et al. (2018a, 2018b)
	AFRCWHEAT2	0.44	–	–	Yes	–	Yes	–	–	P-R	BLL	–	Yes	–	Weir et al. (1984)
	CropSyst	0.45	–	–	–	–	Yes	–	–	RUE/TE	BLC	Yes	–	–	Stockle et al. (1994)
	DAISY	0.60	–	–	Yes	–	Yes	–	–	GP	BLL	–	Yes	–	Hansen et al. (1991)
	DSSAT-CERES-Wheat	0.59	–	–	–	–	Yes	–	–	RUE	BLC	Yes	–	–	Ritchie and Otter (1985)
	DSSAT-CROPSIM	0.65	–	–	Yes	Yes	Yes	–	–	RUE(PAR)	BLC	Yes	–	–	Hunt and Pararajasingham (1995)
	EPIC	0.65	–	–	–	–	Yes	–	–	RUE	BLC	Yes	–	–	Williams et al. (1989)
	FASSET	0.44	–	–	–	–	Yes	–	–	RUE	BLC	Yes	–	–	Olesen et al. (2002)
	GLAM	0.50	–	–	Yes	–	Yes	–	–	TE	BLC	Yes	–	–	Challinor et al. (2004)
	HERMES / MONICA	0.80	–	–		–	Yes	–	–	P-R	BLC	Yes	–	–	Kersebaum (2011)
	InfoCrop	0.60	–	–	Yes	Yes	Yes	–	–	RUE	BLC	Yes	–	–	Aggarwal et al. (2006)
	LINTUL	0.60	–	–	–	–	Yes	–	–	RUE	BLC	Yes	–	–	van Oijen et al. (2004)
	LPjML	0.50	–	–	–	–	–	Yes	–	P-R	BLC	Yes	–	–	Bondeau et al. (2007)
	MCWLA	0.50	–	–	Yes	–	Yes	–	–	P-R	BLC	Yes	–	–	Tao et al. (2009)
	NWheat	0.60–0.7^e^	–	–	–	–	Yes	–	–	RUE	BLC	Yes	–	–	Keating et al. (2003)
	SALUS	0.37–0.67^f^	–	–	–	–	Yes	–	–	RUE	BLC	Yes	–	–	Dzotsi et al. (2013)
	Sirius (v99)	0.45	–	–	✓	–	Yes	–	–	RUE(*f*)	BLC	Yes	–	–	Jamieson et al. (1998)
	*SiriusQuality*	0.45	–	–	✓	–	Yes	–	–	RUE(*f*)	BLL	Yes	–	–	Martre et al. (2006)
	SSM-Wheat	0.65	–	–	–	–	Yes	–	–	RUE	BLC	Yes	–	–	Soltani et al. (2013)
	STICS (v9)	0.50	–	–	Yes	–	Yes	–	–	RUE(PAR)	BLC	Yes	–	–	Brisson et al. (1998)
Spherical	SUCROS97	–	Eq. 10	0.6	–	–	–	Yes	Yes	P-R	SSPGI	–	–	3	Goudriaan and Van Laar (1994)
	SPASS	–	Eq. 8	0.8	Yes	Yes	Yes	–	–	P-R	SSPGI	–	Yes	–	Wang (1997)
	WOFOST	–	Eq. 10	0.6	–	–	–	Yes	Yes	P-R	SSPGI	–	–	3	Supit et al. (1994)
	WHEATGROW	–	Eq. 10	0.67	–	–	–	Yes	Yes	P-R	BLC	–	Yes	–	Liu (2000); Liu et al. (2001)
Average	GECROS	–	Eq. 7	Eq. 23	Yes	Yes	–	Yes	–	P-R	SSPGI	–	–	5	Yin and van Laar (2005)

a GP, gross photosynthesis; P-R, gross photosynthesis–respiration; RUE(*f*), radiation use efficiency approach modified by the fraction of diffuse irradiance; RUE(PAR), radiation use efficiency approach modified by the amount of PAR; TE, transpiration use efficiency approach.

b BLL, big-leaf layer; BLC; big-leaf canopy; SSPGI, sun/shade spatial gaussian integration.

c Three-points (3) or five-points (5) Gaussian integration.

d The light extinction coefficient increases after the beginning of stem extension and until flag leaf ligule following Equation 4.

e The light extinction coefficient increases after anthesis from 0.6 to 0.7.

f The light extinction coefficient is a function of sowing density and row spacing (Eq. 3). The range reported here is for sowing density varying from 100 to 400 seed m^−2^ and row spacing varying from 0.15 to 0.35 m.

The FIPAR models can be grouped into several categories mainly depending on the assumptions used to model *K* (Eq. 2). Below, we first review the models used for direct illumination conditions. Description of variables and parameters of the FIPAR models is listed in Table 3.

**Table 3:** Name, unit, and definition of variables and parameters of the FIPAR models

Name	Unit	Definition
$C_{\mathrm{GAI}}$	–	Clumping factor estimated using GAI
$C_{\mathrm{sph}}$	–	Clumping factor with spherical distribution
*d*	m^−2^	Plant density
f	–	Fraction of diffuse PAR to the total incident PAR
FIPAR	–	Fraction of incident PAR intercepted by the canopy
${\mathrm{FIPAR}}^{\mathrm{dir}}$	–	Fraction of intercepted direct PAR
${\mathrm{FIPAR}}^{\mathrm{dif}}$	–	Fraction of intercepted diffuse PAR
${\mathrm{FIPAR}}_{\mathrm{day}}$	–	Daily value of the fraction of IPAR
FLN	Leaf number	Final number of leaves on the main stem
g	–	Inclination angle distribution function
G	–	Projection function
$G_{\mathrm{avr}}$	–	Projection function assuming that all the green surfaces (leaves, stem and ears) have the same inclination angle and a uniform azimuthal distribution
GAI	m^2^ (leaf) m^−2^ (ground)	GAI
${\mathrm{GAI}}_{\mathrm{eff}}$	m^2^ (leaf) m^−2^ (ground)	Effective GAI
${\mathrm{GAI}}_{\mathrm{mes}}$	m^2^ (leaf) m^−2^ (ground)	Measured GAI
HS	leaf number	Haun stage (decimal mainstem leaf number)
$K_{\mathrm{avr}}^{\mathrm{dir}}$	–	Extinction coefficient with average distribution for direct PAR
$K_{\mathrm{cst}}^{}$	–	Constant extinction coefficient
$K_{\mathrm{cst}}^{\mathrm{ds}}$	–	Constant extinction coefficient accounting for plant density and row spacing
$K_{\mathrm{cst}}^{\mathrm{HS}}$	–	Constant extinction coefficient accounting for the increment after stem elongation
$K_{\mathrm{ell}}^{\mathrm{dir}}$	–	Extinction coefficient with ellipsoidal distribution for direct PAR
$K_{\mathrm{ell}}^{C}$	–	Extinction coefficient with spherical distribution and considering clumping effect
$K_{\mathrm{mes}}^{\mathrm{dif}}$	–	Measured extinction coefficient for the diffuse incoming PAR
$K_{\mathrm{sph}}^{\mathrm{dir}}$	–	Extinction coefficient with spherical distribution for direct PAR
$K_{\mathrm{sph}}^{C}$	–	Extinction coefficient with spherical distribution and considering clumping effect
PAR	W m^−2^	Incoming photosynthetically active radiation
*s*	m	Row spacing
$\beta_{b}$	Radian	Half azimuth range for which the upper side of a leaf is illuminated
β	Radian	Sun elevation angle
τ	–	Fraction of PAR transmitted to the ground level
Λ	–	Normalized ellipse area
σ	–	Scattering coefficient of leaves for PAR
θ	Radian	Inclination angle of the green surfaces in the canopy
$\bar{\theta }$	Radian	Average inclination angle of the green surfaces in the canopy
ω	Leaf number	Haun stage at which $K_{\mathrm{cst}}^{\mathrm{HS}}$ starts to increases in the $K_{\mathrm{cst}}^{\mathrm{HS}}$ model
χ	–	Eccentricity of the ellipsoidal leaf inclination distribution

### Models of light interception for direct illumination conditions

#### Constant light extinction coefficient

The simplest and most popular FIPAR model in wheat CGMs (Table 2) assumes a constant light extinction coefficient ($K_{\mathrm{cst}}^{}$) and FIPAR is calculated using Equation 1. By definition, $K_{\mathrm{cst}}^{}$ is assumed to be valid for any light direction and therefore applies to both direct and diffuse incident PAR. The values of $K_{\mathrm{cst}}^{}$ used in wheat GCMs range from 0.44 to 0.80 (Table 2), with a median value of 0.52, that is, close to that calculated from field measurements (Table 1).

Some adaptations have been proposed to account for the impact of plant arrangement or ontogenic changes in canopy structure on $K_{\mathrm{cst}}^{}$. In the System Approach to Land Use Sustainability (SALUS), the sowing pattern is accounted for by using an empirical relationship derived from experimental observations (Dzotsi et al., 2013):

(3) $$K_{\mathrm{cst}}^{\mathrm{ds}}=1.5-0.768{\left(d{\times s}^{2}\right)}^{0.1}$$

where d is the plant density (plants m^−2^) and s is the row spacing (m). $K_{\mathrm{cst}}^{\mathrm{ds}}$ varies between 0.37 and 0.67 for d ranging between 100 and 400 plants m^−2^ and s ranging from 0.15 to 0.35 m, with $K_{\mathrm{cst}}^{\mathrm{ds}}$ decreasing with increasing row spacing and plant density.

In the latest version of APSIM Wheat (Brown et al., 2018a, 2018b), $K_{\mathrm{cst}}^{}$ increases during the stem extension period because of the change in leaf inclination, but probably also because the stem and ear area were not explicitly accounted for:

(4) KcstHS=0.5, HS≤ω0.51+0.2HS-ωFLN-ω, HS>ω

where HS is the Haun stage (Haun, 1973) of the main stem, FLN is the final number of leaves on the main stem, and ω is the Haun stage at which $K_{\mathrm{cst}}^{\mathrm{HS}}$ starts to increase. ω is given by:

(5) ω=0.973×FLN-0.777

For the same reasons as for APSIM Wheat, in Nwheat model, $K_{\mathrm{cst}}^{}$ changes from 0.6 before anthesis to 0.7 after anthesis (Keating et al., 2003).

### Average surface inclination

The average surface inclination angle over the whole canopy (θ) can be calculated as the average zenith angle of all the green surface elements weighted by their area. This includes green leaves, visible green sheath parts, internodes, and ears. At the canopy level, assuming that all the green surfaces have the same inclination angle and a uniform azimuthal distribution, the projection function, $G_{\mathrm{avr}}\left(\beta ,\;\theta \right)$ is given by (Goudriaan, 1988):

(6) Gavrβ, θ={sin⁡β×cos⁡ θ, β≥ θ2πsin⁡β×cos⁡ θ arcsintan⁡βtan⁡ θ+sin2⁡ θ-sin2⁡β,β<θ

*K* can then be calculated as:

(7) $$K_{\mathrm{avr}}^{\mathrm{dir}}=\frac{G_{\mathrm{avr}}\left(\beta ,\;\theta \right)}{\sin\beta }$$

This formulation of the projection function is used in GECROS (Yin and van Laar, 2005) with a default value of θ for wheat of 50°.

### Spherical surface inclination distribution

In the simple case where the surfaces of all green organs are distributed as the facets around a sphere, G = 0.5 (Goudriaan, 1977, 1988). In this case, *K* is given by:

(8) $$K_{\mathrm{sph}}^{\mathrm{dir}}=\frac{0.5}{\sin\beta }$$

In the SPASS model, *K* is calculated using Equation 8 (Wang, 1997).

Goudriaan and Van Laar (1994) proposed an adaptation of the spherical model to better match field observations. They proposed to account for the departure between observed FAPAR and the one computed assuming a spherical surface angle distribution by using a correction factor ($C_{\mathrm{sph}}$), calculated as the measured *K* for diffuse PAR ($K_{\mathrm{meas}}^{\mathrm{diff}}$) relative to the theoretical value for spherical surface angle distribution.$\;C_{\mathrm{sph}}$ is given by:

(9) $$C_{\mathrm{sph}}=\frac{K_{\mathrm{meas}}^{\mathrm{dif}}}{0.8\sqrt{1-\sigma }}$$

where σ is the scattering coefficient of leaves in the PAR domain. This approach is used in SUCROS (Goudriaan and Van Laar, 1994), WOFOST (Goudriaan and Van Laar, 1994), and WheatGrow (Liu, 2000). In these GCMs, *K* for direct light is thus given by:

(10) $$K_{\mathrm{sph}}^{C}=C_{\mathrm{sph}}\times K_{\mathrm{sph}}^{\mathrm{dir}}$$

In SUCROS and WOFOST, $K_{\mathrm{sph}}^{\mathrm{dif}}$ is considered as constant and is set equal to 0.6 for wheat (Goudriaan and Van Laar, 1994). Thus $C_{\mathrm{sph}}=0.75$ and $K_{\mathrm{sph}}^{C}=0.375/\sin\beta$. In WheatGrow, $K_{\mathrm{sph}}^{\mathrm{dif}}$ =0.67, and thus $K_{\mathrm{sph}}^{C}=0.42/\sin\beta$ (Liu, 2000). Here, we used the value of $C_{\mathrm{sph}}$ proposed by Goudriaan and Van Laar (1994).

### Ellipsoidal surface inclination distribution

The spherical distribution can be generalized by considering that the orientation of the surfaces of the green organs is distributed as the facets around an ellipsoid of revolution with a vertical rotation axis (Campbell, 1986). The ellipsoid is characterized by its eccentricity, χ, that is, the ratio between its vertical and horizontal diameters. By varying χ, the distribution of angles simulates planophile up to erectophile leaf stature, as well as the spherical distribution when χ = 1.0. The ellipsoidal function is a good trade-off between simplicity and flexibility in characterizing the distribution of surface inclination at the canopy scale (Weiss et al., 2004; Wang et al., 2007). Nevertheless, it has never been evaluated over wheat canopies due to the lack of leaf curvature measurements, which require considerable field work.

The analytical expression of the ellipsoidal inclination distribution function, $g\left(\theta \right)$, is given by (Campbell, 1990):

(11) $$g\left(\theta \right)=\frac{2{\times \chi }^{3}\times \sin\theta }{\Lambda {\left(\cos^{2}\theta +\chi^{2}\times \sin^{2}\theta \right)}^{2}}$$

where Λ is a normalized ellipse area, approximated by:

(12) $$\Lambda =\;\chi +{1.774(\chi +1.182)}^{-0.733}$$

*χ* is related to the average inclination angle at the canopy scale ($\bar{\theta }$) through an empirical equation:

(13) $$\chi ={\left(\frac{\bar{\theta }}{9.65}\right)}^{-0.6061}-3$$

Following the approach proposed by Verhoef (1998), *K* of any leaf angle distribution is computed as the integral over the whole range of leaf inclination (0 < θ < π/2) of $K\left(\beta ,\theta \right)$ weighted by the corresponding frequency, $g\left(\theta \right)$. Since the analytical solution is not straightforward, a discrete approximation was proposed by Verhoef (1998) for a sample of n inclination angles:

(14) $$K_{\mathrm{ell}}^{\mathrm{dir}}=\frac{\sum_{i=1}^{n}g\left(\theta_{i}\right)\times K\left(\beta ,\theta_{i}\right)}{\sum_{i=1}^{n}g\left(\theta_{i}\right)}$$

According to Verhoef (1998), it is sufficient if the inclination angle is discretized into 13 classes (5°, 15°, 25°, 35°, 45°, 55°, 65°, 75°, 81°, 83°, 85°, 87°, and 89°), expressed in radians in Equations 14 and 15. $K_{\mathrm{ell}}^{}(\beta ,\theta_{i})$ for inclination angle $\theta_{i}$ writes:

(15) $$K_{\mathrm{ell}}^{\mathrm{dir}}=\frac{2}{\pi }\left[\left(\beta_{b}-\frac{2}{\pi }\right)\cos\theta_{i}+\frac{\sin\beta_{b}\times \sin\theta_{i}}{\tan\beta }\right]$$

with,

(16) $$\beta_{b}=\left\{\begin{array}{cc} \pi ,\; & \theta_{i}<\beta \\ \mathrm{arcos}\left(-\frac{\tan\beta }{\tan\theta_{i}}\right), & \theta_{i}\geq \beta \end{array}\right.$$

where $\beta_{b}$ (radian) is the transition angle, which is half of the azimuth range for which the upper side of a leaf is illuminated.

Alternatively, $K_{\mathrm{ell}}^{}$ can be approximated by (Campbell and Norman, 2012):

(17) $$K_{\mathrm{ell}}^{\mathrm{dir}}=\frac{\sqrt{\chi^{2}+\cot^{2}\beta }}{\chi +{1.774(\chi +1.182)}^{-0.733}}$$

In this work, we used Equation 17 to calculate $K_{\mathrm{ell}}^{\mathrm{dir}}.$

The calculation of $C_{\mathrm{sph}}$ using Equation 9 is an approximation since FIPAR for diffuse radiation calculated from Equation 21 cannot be analytically put in the form of Equation 1 to derive $K_{\mathrm{sph}}^{\mathrm{dif}}$. Therefore, Baret et al. (2010) proposed to estimate the clumping factor as the ratio of the effective GAI, estimated from the directional variation of FIPAR (${\mathrm{GAI}}_{\mathrm{eff}}$), to the actual GAI from destructive measurement (${\mathrm{GAI}}_{\mathrm{mes}}$):

(18) $$C_{\mathrm{GAI}}=\frac{{\mathrm{GAI}}_{\mathrm{eff}}}{{\mathrm{GAI}}_{\mathrm{mes}}}$$

Where ${\mathrm{GAI}}_{\mathrm{eff}}$ is calculated following Miller (1967):

(19) $${\mathrm{GAI}}_{\mathrm{eff}}\left(\beta \right)=2\int_{0}^{\pi /2}-\ln\left(1-\mathrm{FIPAR}\left(\beta \right)\right)\cos\beta \times \sin\beta \times d\beta$$

We use the approach of Baret et al. (2010) to take into account leaf clumping in the ellipsoidal surface inclination distribution model. The extinction coefficient for an ellipsoidal surface inclination distribution accounting for leaf clumping ($K_{\mathrm{ell}}^{C}$) is then given by:

(20) $$K_{\mathrm{ell}}^{C}={C_{\mathrm{GAI}}\times K}_{\mathrm{ell}}^{\mathrm{dir}}$$

### Accounting for diffuse radiation

At a given time of the day, the incident radiation is coming both from the sun direction and from the light scattered in the atmosphere, which is generally assumed to be isotropic. The total amount of radiation intercepted at a given time t of the day is thus given by:

(21) $$\mathrm{FIPAR}=f\left(t\right)\times {\mathrm{FIPAR}}^{\mathrm{dif}}+\left(1-f\left(t\right)\right)\times {\mathrm{FIPAR}}^{\mathrm{dir}}\left(\beta \left(t\right)\right)$$

where $f\left(t\right)$ is the fraction of diffuse radiation in the PAR domain, and ${\mathrm{FIPAR}}^{\mathrm{dir}}$ and ${\mathrm{FIPAR}}^{\mathrm{dif}}\;$are the FIPAR for direct and diffuse radiation, respectively. The daily value of FIPAR (${\mathrm{FIPAR}}_{\mathrm{day}}^{}$) can then be calculated by integrating the instantaneous values as follows:

(22) $${\mathrm{FIPAR}}_{\mathrm{day}}^{}=\frac{\sum_{\mathrm{sunset}}^{\mathrm{sunrise}}\mathrm{FIPAR}\times \mathrm{PAR}\;}{\sum_{\mathrm{sunset}}^{\mathrm{sunrise}}\mathrm{PAR}}$$

When FIPAR does not depend on β, as for the $K_{\mathrm{cst}}^{}$ model, the instantaneous value of FIPAR is independent of the diffuse fraction since $\mathrm{FIPAR}\;=\;{\mathrm{FIPAR}}^{\mathrm{dif}}\;=\;{\mathrm{FIPAR}}^{\mathrm{dir}}$. The spherical inclination distribution and average inclination models use a different value of *K* for ${\mathrm{FIPAR}}^{\mathrm{dif}}$ and ${\mathrm{FIPAR}}^{\mathrm{dir}}$. The CGMs that use a spherical inclination distribution model use a constant value of *K* for diffuse PAR (Table 2). Here we used the value of 0.6 proposed by Goudriaan and Van Laar (1994). In GECROS, which uses an average inclination model at the canopy scale, *K* for diffuse radiation ($K_{\mathrm{avr}}^{\mathrm{dif}}$) is calculated following Goudriaan (1988) and is given by:

(23) $$K_{\mathrm{avr}}^{\mathrm{dif}}=-\left(1/\mathrm{GAI}\right)\times ln(0.178\times e^{\left(-K_{b15}\times \mathrm{GAI}\right)}+0.514\times e^{\left(-K_{b45}\times \mathrm{GAI}\right)}+0.308\times e^{\left(-K_{b75}\times \mathrm{GAI}\right)})$$

where $K_{b15}$, $K_{b45}$, ${\mathrm{and}\;K}_{b75}$ are the extinction coefficients calculated using Equation (7) for β equals 15°, 45°, and 75°, respectively. The weights 0.178, 0.514, and 0.308 represent the contributions from the 30° classes (0–30°, 30–60°, and 60–90°) of a standard overcast sky.

For the ellipsoidal inclination distribution model, we calculated ${\mathrm{FIPAR}}^{\mathrm{dif}}$ as the integral over the hemisphere of the directional ${\mathrm{FIPAR}}^{\mathrm{dir}}$:

(24) $${\mathrm{FIPAR}}^{\mathrm{dif}}=2\int_{0}^{\pi /2}{\mathrm{FIPAR}}^{\mathrm{dir}}\left(\beta \right)\times \cos\beta \times \sin\beta \times d\beta$$

### Canopy elements considered as intercepting area

As already discussed, GAI should be considered in Equation 1, including both one-sided area of flat green organs (leaves) and half the developed area of nonflat green organs (stems or ears) (Lang, 1991; Chen and Black, 1992). However, *K* is often estimated using measurements of FIPAR and the corresponding area of some of the crop elements (Table 1). All the models consider the leaf lamina, but only few consider the area of stems and ears (Table 2), although this may represent a substantial fraction of GAI (Weiss et al., 2001). Some models such as APSIM wheat and Nwheat compensate for the fact that the stems and ears are not explicitly accounted for by increasing *K* after the onset of stem extension (APSIM wheat) or after anthesis (Nwheat). However, for the other models, no obvious trend is observed between the value of the extinction coefficient and the elements accounted for in the GAI (Table 2), similarly to what was found from the ground measurements (Table 1). In the case of the SUCROS, WOFOST, and WheatGrow, which do not account for stems and ears area (Table 2), the empirically adjusted clumping factor may partly compensate for this approximation.

## Results

### The ellipsoidal inclination distribution faithfully represents the actual distribution of surface inclination in wheat

The 3D canopies reconstructed from the digitalization of the field-grown plants were used to extract the inclination angle distribution, corresponding to the distribution of the zenith angle of all green elements in the canopy. The variance of $\bar{\theta }$ over treatments was much larger at GS31 than at GS39 (Supplemental Table S2). At both growth stages, $\bar{\theta }$ varied more between cultivars than with row spacing. However, both cultivar and row spacing effects on $\bar{\theta }$ were not statistically significant (*P* > 0.01; Supplemental Table S3). The canopies were more erectophile at GS39 as compared to GS31, mostly because $\bar{\;\theta }$ of leaf elements increased from 39° at GS31 to 63° at GS39 (Supplemental Table S2; Supplemental Figure S3). Stems were also more erectophile at GS39 ($\bar{\theta }$ = 84°) than at GS31 ($\bar{\theta }\;$ = 75°).

According to Equation 13 (solving the equation with χ = 1), $\bar{\theta }$ equals 56° for spherical leaf angle distribution. At GS31, Caphorn, the most erectophile of the five studied cultivars, was the cultivar with $\bar{\theta }$ closest to that of a spherical distribution (χ = 1.14; Table 4). For the four other cultivars, $\bar{\theta }$ was lower than 56° (Table 4) and χ ranged from 1.40 (for Apache) to 2.07 (for Maxwell). At GS39, $\bar{\theta }$ was close to 70° (i.e. χ = 0.50) for the four cultivars (Table 4) and was thus much higher than the value corresponding to a spherical distribution.

**Table 4:** Average inclination angle of green elements in the canopy measured at the beginning of stem extension (GS31) and flag leaf ligulation (GS39) for five winter wheat cultivars grown in the field with standard (17.5 cm, SS) and double (35 cm, DS) row spacing

Growth Stage	Average inclination angle of green elements (°)									
	Apache		Caphorn		Maxwell		Renan		Soissons		
	SS	DS	SS	DS	SS	DS	SS	DS	SS	DS	
31	48 ± 16	51 ± 16	53 ± 16	52 ± 16	38 ± 16	41 ± 14	39 ± 16	49 ± 17	43 ± 16	48 ± 16	
39	69 ± 12	65 ± 15	73 ± 9	72 ± 9	69 ± 11	72 ± 11	70 ± 12	69 ± 13	71 ± 11	67 ± 12	

Data are mean ± 1 sd.

**Figure 1:**
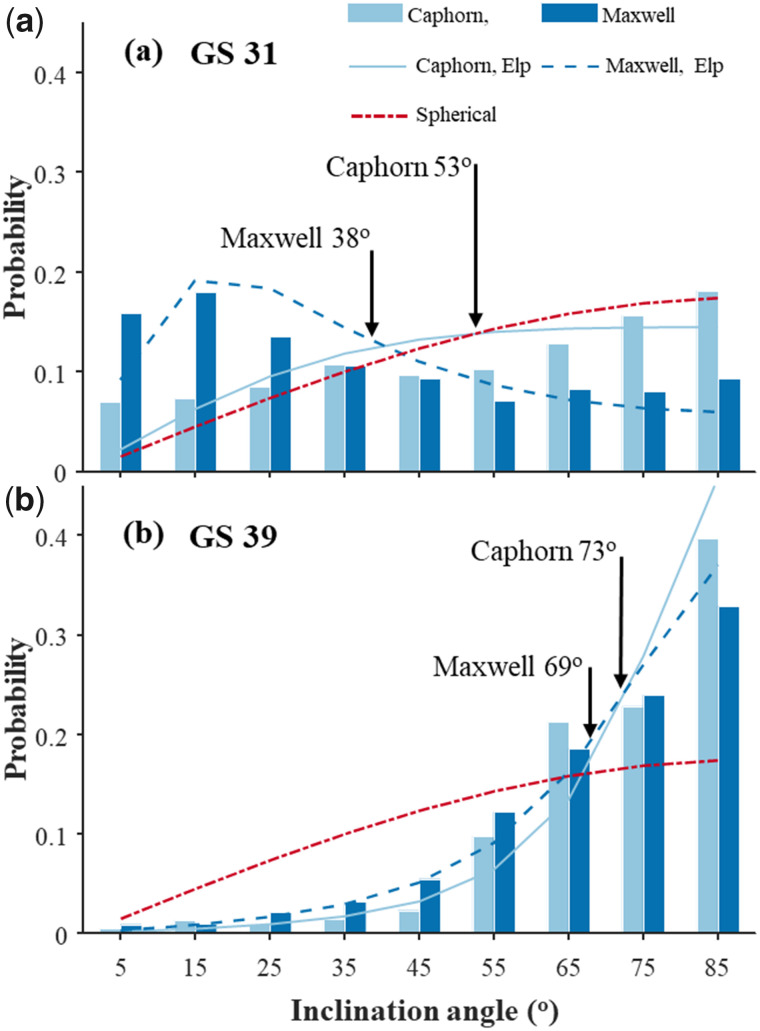
Distributions of inclination angle of canopies’ green elements. Measured (bars) distributions of inclination angle of canopies’ green elements at the beginning of stem extension (GS31, A) and flag leaf ligulation (GS39, B) for the winter wheat cultivars Caphorn (light blue) and Maxwell (dark blue) grown in the field with standard row spacing. Solid and dashed blue lines are the distributions of canopy inclination angles calculated with the ellipsoidal model for Caphorn and Maxwell, respectively. Dot-dashed red lines are the distributions of canopy inclination angles calculated with the spherical model. Vertical arrows indicate the average angle for each growth stage and cultivar.

For all the cultivars, growth stages, and row spacing treatments, the distribution of inclination was not significantly different from an ellipsoidal distribution (all *P* > 0.01). Conversely, in all cases the distribution of inclination was significantly different from a spherical distribution (all *P* < 0.01). When $\bar{\theta }\;$is ˃80^°^, the ellipsoidal distribution tended to underestimate the corresponding probability at GS31, while overestimating it at GS39 (Figure 1).

### Increasing row spacing decreases the clumping factor

**Figure 2:**
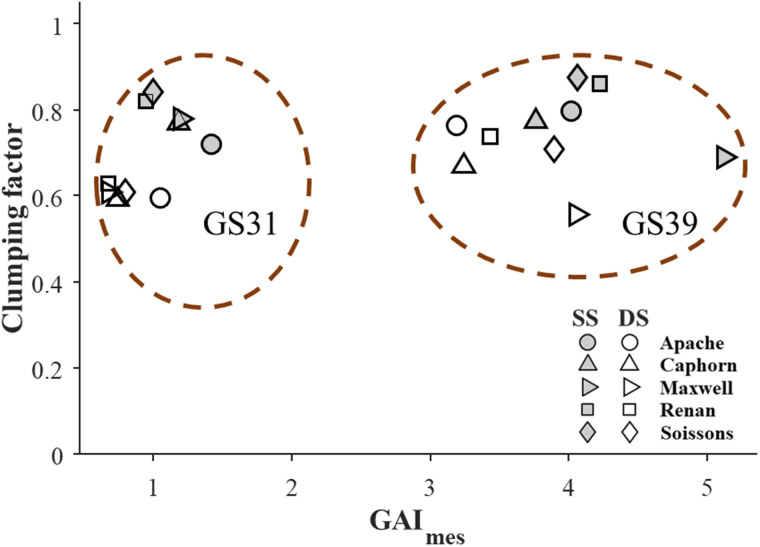
Clumping factor versus measured GAI GAI_mes_. The clumping factor was calculated using Equation 18 and the 3D reconstructions of canopy structures at the beginning of stem extension (GS31) and flag leaf ligule (GS39) for five winter wheat cultivars grown in the field with SS (open symbols) and DS (closed symbols) row spacing. The two red dashed circles indicate points from stage GS31 and GS39, respectively.

The clumping factor ($C_{\mathrm{GAI}}$) was computed over the reconstructed 3D wheat canopies by using Equation 18. At GS31, for the five cultivars $C_{\mathrm{GAI}}$ was on average 18% higher for the standard ($C_{\mathrm{GAI}}$ = 0.79) than for the double row spacing treatment ($C_{\mathrm{GAI}}$ = 0.67, Figure 2). At GS39, $C_{\mathrm{GAI}}$ was always slightly lower for the double spacing as compared to the standard spacing. For standard row spacing, $C_{\mathrm{GAI}}$ values were similar for the two growth stages. Conversely, for double-row spacing, $C_{\mathrm{GAI}}$ was on average 17% higher at GS39 than at GS31, except for Maxwell.

### Comparison between FIPAR models

**Figure 3:**
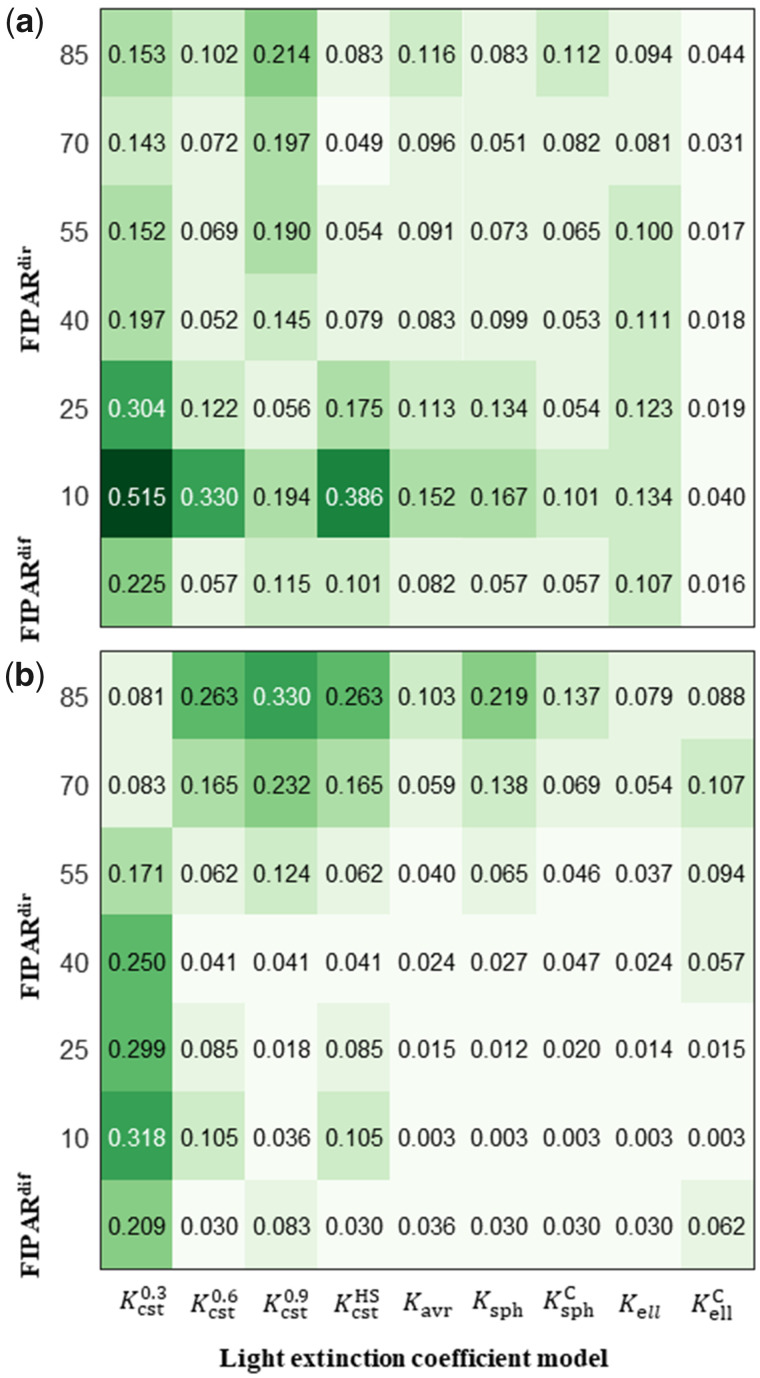
Comparison among light extinction coefficient models. RMSE for the fraction of intercepted diffuse (${\mathrm{FIPAR}}^{\mathrm{dif}}$) and direct (${\mathrm{FIPAR}}^{\mathrm{dir}}$) PAR, calculated with different light extinction coefficient models at the beginning of stem extension (GS31, A) and at flag leaf ligulation (GS39, B). ${\mathrm{FIPAR}}^{\mathrm{dif}}$ and ${\mathrm{FIPAR}}^{\mathrm{dir}}$ were calculated from the 3D reconstructed canopies and illumination conditions measured in Grignon in 2013. The RMSE for ${\mathrm{FIPAR}}^{\mathrm{dir}}$ was calculated for solar elevations varying from 10° to 85°. Light extinction models are: constant values ($K_{\mathrm{cst}}^{0.3},\;K_{\mathrm{cst}}^{0.6}$, $K_{\mathrm{cst}}^{0.9}$), constant values increased during the stem extension period ($K_{\mathrm{cst}}^{\mathrm{HS}}$), average leaf inclination angle ($K_{\mathrm{avr}}^{}$), spherical leaf inclination angle distribution ($K_{\mathrm{sph}}^{}$), spherical leaf inclination angle distribution accounting for clumping ($K_{\mathrm{sph}}^{C}$), ellipsoidal leaf inclination angle distribution ($K_{\mathrm{ell}}^{}$), and ellipsoidal leaf inclination angle distribution accounting for clumping ($K_{\mathrm{ell}}^{C}$). Data are RMSE calculated for the five cultivars and two-row spacing treatments. The greenness of the background color increases with the corresponding RMSE value.

For ${\mathrm{FIPAR}}^{\mathrm{dir}}$, at both GS31 and GS39 the ellipsoidal model outperformed the other models over all solar angles (Figure 3). At GS31, the root mean squared error (RMSE) for ${\mathrm{FIPAR}}^{\mathrm{dir}}$ was reduced by 37% when leaf clumping was accounted for in the ellipsoidal model (Figure 3A). The spherical models ($K_{\mathrm{sph}}^{}$ and $K_{\mathrm{sph}}^{C}$) and average leaf inclination angle ($K_{\mathrm{avr}}^{}$) provided reasonable performances, while the models with constant light extinction coefficients ($K_{\mathrm{cst}}^{}$) substantially degraded the accuracy of ${\mathrm{FIPAR}}^{\mathrm{dir}}$ calculation, with 0.013 < RMSE < 0.23 ($K_{\mathrm{cst}}^{0.6}$ > $K_{\mathrm{cst}}^{0.9}$ > $K_{\mathrm{cst}}^{0.3}$). For ${\mathrm{FIPAR}}^{\mathrm{dir}}$, the differences between the models were larger for low solar elevation angles at GS31 (Figure 3A) and for high solar elevation angels at GS39 (Figure 3B). For both growth stages, the differences between the models were the lowest for solar elevation between 40° and 55°. Under diffuse illumination conditions, $K_{\mathrm{ell}}^{C}$ also performed the best at GS31 (Figure 3A), while at GS39 the RMSE was low for several models and $K_{\mathrm{ell}}^{C}$ was not the best model (Figure 3B).

### Sensitivity of FIPAR to canopy structure and illumination conditions

**Figure 4:**
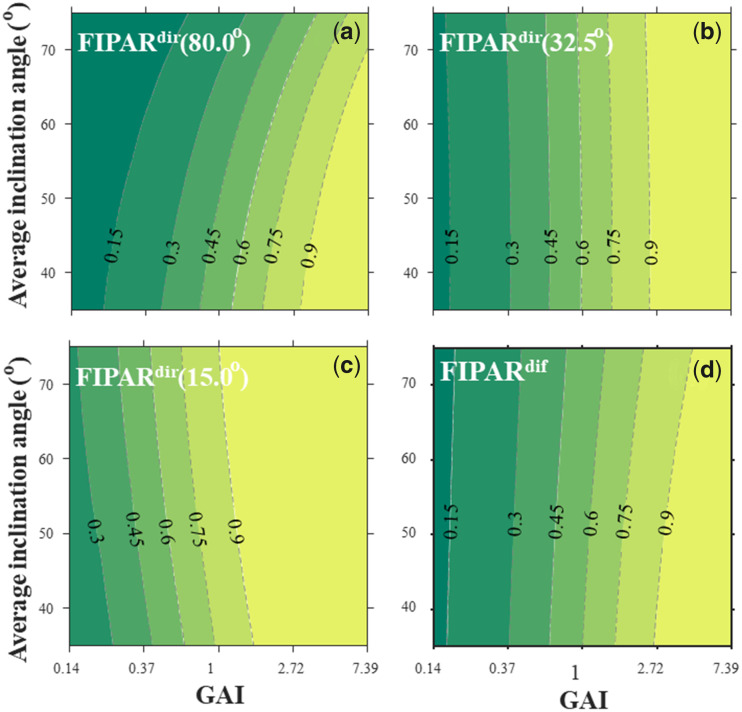
Sensitivity of the fraction of IPAR to various canopy structure. Sensitivity of the fraction of intercepted directs (${\mathrm{FIPAR}}^{\mathrm{dir}};$ A–C) and diffuse (${\mathrm{FIPAR}}^{\mathrm{dif}}$; D) PAR to GAI. ${\mathrm{FIPAR}}^{\mathrm{dir}}$ was calculated for 80^°^ (A), 32.5^°^ (B), and 15^°^ (C) solar elevations. ${\mathrm{FIPAR}}^{\mathrm{dif}}$ and ${\mathrm{FIPAR}}^{\mathrm{dir}}$ were calculated with the ellipsoidal model accounting for clumping (with $C_{\mathrm{GAI}}$ = 0.79). GAI is plotted on a logarithmic scale. The background color varies from dark green to yellow indicates the increase of FIPAR value.

Since we found that $K_{\mathrm{ell}}^{C}$ was the best model for FIPAR, we used it to explore the sensitivity of FIPAR with respect to changes in canopy structure, GAI and $\bar{\theta }$ (Figure 4). Results show that FIPAR increased rapidly with GAI, as expected from the exponential form of Equation 1. ${\mathrm{FIPAR}}^{\mathrm{dir}}$ was insensitive to $\bar{\theta }$ when the sun was at 32.5° elevation (Figure 4B). ${\mathrm{FIPAR}}_{}^{\mathrm{dir}}$ increased with $\bar{\theta }$ for β < 32.5° (Figure 4C), and decreased with $\bar{\theta }$ for β > 32.5°. The influence of $\bar{\theta }$ on ${\mathrm{FIPAR}}^{\mathrm{dif}}$ was low (Figure 4D) because ${\mathrm{FIPAR}}^{\mathrm{dif}}$ was computed by integrating ${\mathrm{FIPAR}}^{\mathrm{dir}}$ over all directions, which put maximum weight on elevations close to 45°, that is, not too far from 32.5°.

**Figure 5:**
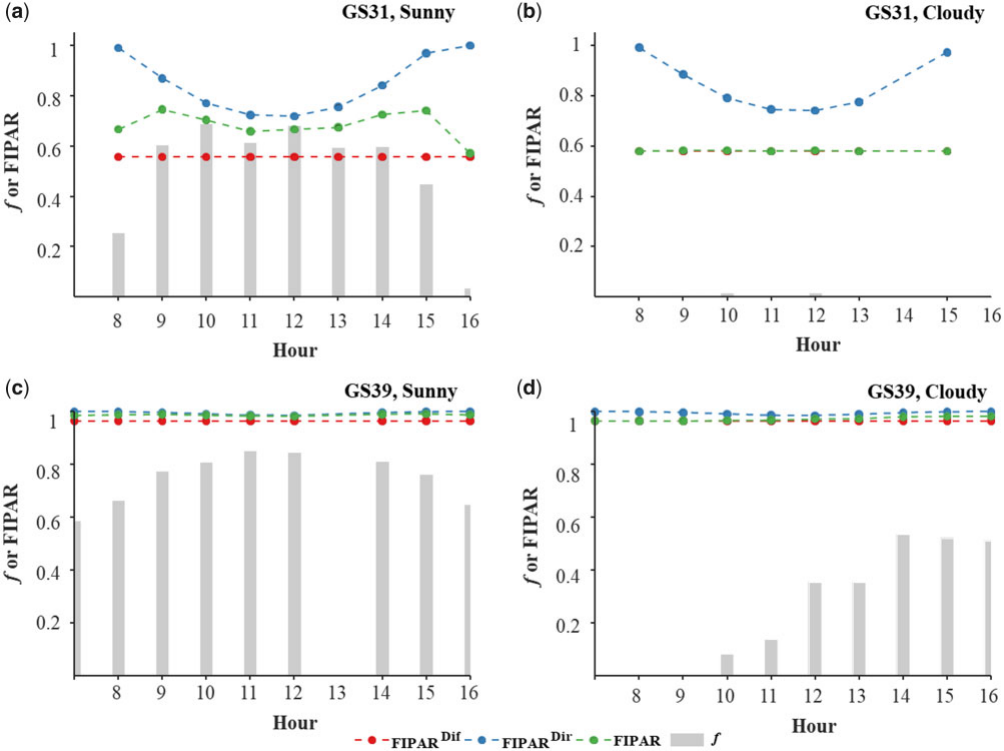
Diurnal course of the fraction of diffuse PAR (f) and the fraction of IPAR (FIPAR). A, C demonstrate under clear while (B) and (D) shows under overcast sky conditions at Grignon, France, calculated using the ellipsoidal model accounting for clumping (with $C_{\mathrm{GAI}}$ = 0.79). The canopy structure corresponds to the cultivar Caphorn at the beginning of stem extension (A and B) and flag leaf ligule (C and D) with standard row spacing.

Since most CGMs have a daily time step (Table 2), it is necessary to integrate ${\mathrm{FIPAR}}^{\mathrm{dir}}$ and ${\mathrm{FIPAR}}^{\mathrm{dif}}$ over the day, according to Equation 22 to get ${\mathrm{FIPAR}}_{\mathrm{day}}^{}$. Simulations showed that, for a given canopy structure, the value of ${\mathrm{FIPAR}}_{\mathrm{day}}^{}$ may change largely, depending on the illumination conditions during the day (Figure 5). At GS31, under clear sky conditions, the solar elevation at solar noon is ∼45° at this period of the year and latitude, and ${\mathrm{FIPAR}}_{\mathrm{day}}^{}$ equals 0.25, while under overcast sky conditions ${\mathrm{FIPAR}}_{\mathrm{day}}^{}$ is very close to ${\mathrm{FIPAR}}^{\mathrm{dif}}$ and is 32% lower than under clear sky conditions.

### Differences between light extinction models over longer time periods

**Figure 6:**
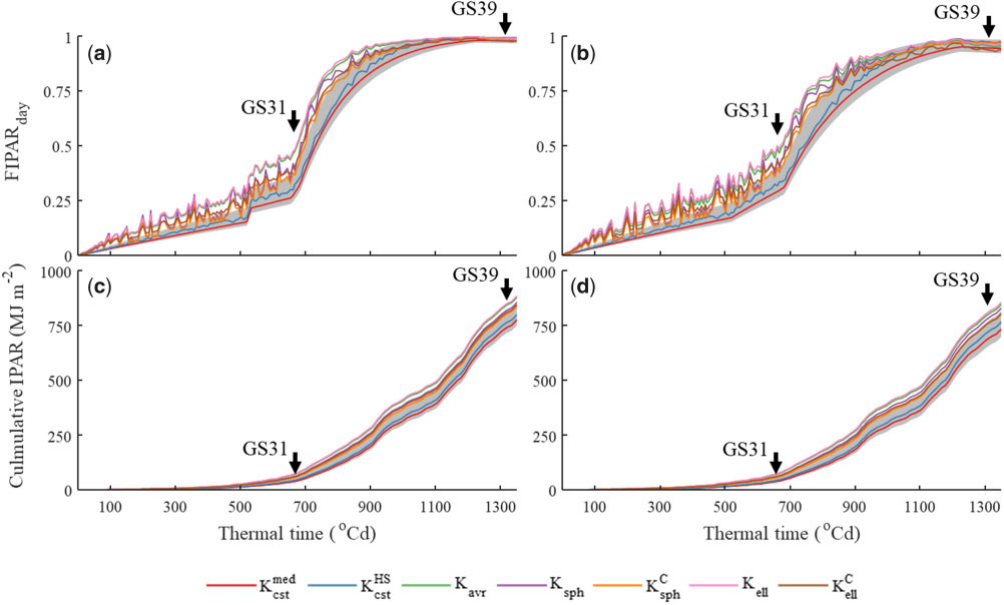
Dynamic of daily fraction of IPAR (FIPAR_day_) and cumulative IPAR. The change of FIPAR_day_ (A and B) and IPAR (C and D) versus thermal time after crop emergence for the winter wheat cultivars Maxwell (A and C) and Caphorn (B and D), calculated with different light extinction models. Light extinction models are: constant values ($K_{\mathrm{cst}}^{\mathrm{med}}$), constant values increased during the stem extension period ($K_{\mathrm{cst}}^{\mathrm{HS}}$), average leaf inclination angle ($K_{\mathrm{avr}}^{}$), spherical leaf inclination angle distribution ($K_{\mathrm{sph}}^{}$), spherical leaf inclination angle distribution accounting for clumping ($K_{\mathrm{sph}}^{C}$), ellipsoidal leaf inclination angle distribution ($K_{\mathrm{ell}}^{}$), and ellipsoidal leaf inclination angle distribution accounting for clumping ($K_{\mathrm{ell}}^{C}$). $K_{\mathrm{cst}}^{\mathrm{med}}$ is the median value for the 22 models that use a constant *K* value (Table 2), and the gray banding shows the 5% and 95% percentile. The canopy structure and weather data correspond to the 2012–2013 experiment in Grignon, France with standard row spacing. Vertical arrows indicate the beginning of stem extension (GS31) and flag leaf ligule (GS39).

The comparison of ${\mathrm{FIPAR}}_{\mathrm{day}}^{}$ simulated with the different FIPAR models in Grignon for Caphorn and Maxwell cultivars with standard row spacing showed that most differences between models appeared during the early vegetative growth period (Figure 6, A and B). The models that considered solar elevation and diffuse and direct PAR (all models except $K_{\mathrm{cst}}^{}$) showed day-to-day ${\mathrm{FIPAR}}_{\mathrm{day}}^{}$ variations, demonstrating their sensitivity to the illumination conditions. Compared with $K_{\mathrm{ell}}^{}$, $K_{\mathrm{sph}}^{}$ underestimated ${\mathrm{FIPAR}}_{\mathrm{day}}^{}$ by ∼12% and 13% for Maxwell and Caphorn, respectively. ${\mathrm{FIPAR}}_{\mathrm{day}}^{}$ was ∼13% and 6% relatively lower when leaf clumping was accounted for in the $K_{\mathrm{ell}}^{}$ and $K_{\mathrm{sph}}^{}$ models, respectively. The uncertainty in ${\mathrm{FIPAR}}_{\mathrm{day}}^{}$ between the CGMs that use the $K_{\mathrm{cst}}^{}$ model was ∼15% on average (gray banding in Figure 6). The $K_{\mathrm{cst}}^{\mathrm{med}}$ model underestimated ${\mathrm{FIPAR}}_{\mathrm{day}}^{}$ by 28% and 29% for Maxwell and Caphorn compared with the $K_{\mathrm{ell}}^{C}$ model. The performance of the $K_{\mathrm{avr}}^{}$ model was close to the $K_{\mathrm{ell}}^{}$ model, with a relative difference of 2% and 3% for Maxwell and Caphorn, respectively. Compared with the $K_{\mathrm{ell}}^{C}$ model, the $K_{\mathrm{cst}}^{\mathrm{med}}$ model underestimated the cumulative intercepted PAR (IPAR) by 35% and 10% at GS31 and GS39, respectively (Figure 6, C and D), while the $K_{\mathrm{sph}}^{}$ and $K_{\mathrm{avr}}^{}\;$models overestimated it by 2% and 16% at GS31 and by 2% and 5% at GS39, respectively.

### Uncertainty in crop growth and grain yield simulations due to light extinction models

**Figure 7:**
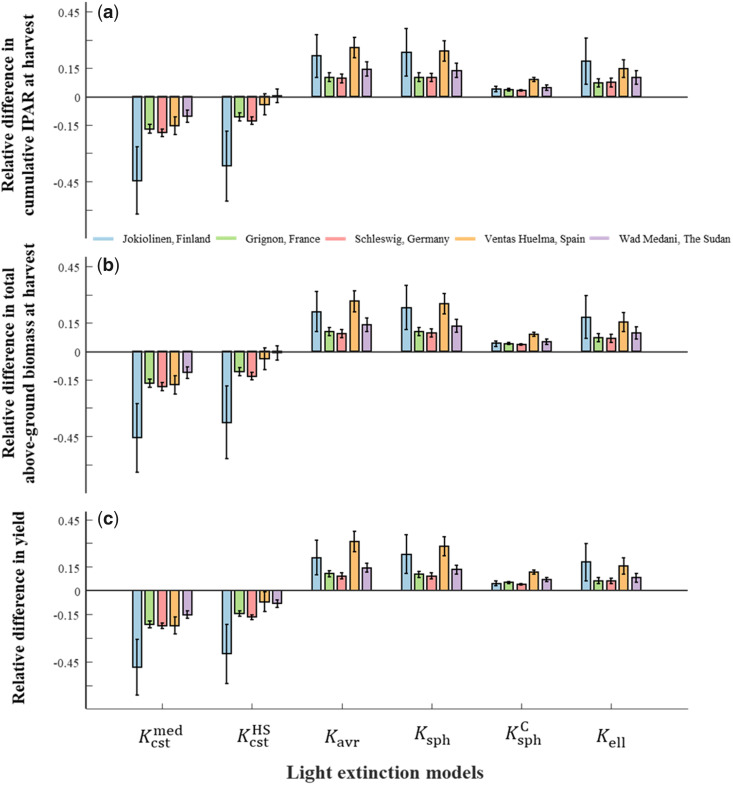
Uncertainty in simulated cumulative IPAR, total above-ground biomass, and grain yield at harvest due to different light extinction models at five sites spanning the range of latitudes at which wheat is grown. Relative differences in the three variables between the different models and the model with ellipsoidal leaf inclination angle distribution accounting for clumping ($K_{\mathrm{ell}}^{C}$) considered as reference are shown. Light extinction models are: constant light extinction coefficient ($K_{\mathrm{cst}}^{\mathrm{med}}$), constant values increased during the stem extension period ($K_{\mathrm{cst}}^{\mathrm{HS}}$), average leaf inclination angle ($K_{\mathrm{avr}}^{}$), spherical leaf inclination angle distribution ($K_{\mathrm{sph}}^{}$), spherical leaf inclination angle distribution accounting for clumping ($K_{\mathrm{sph}}^{C}$), ellipsoidal leaf inclination angle distribution ($K_{\mathrm{ell}}^{}$). $K_{\mathrm{cst}}^{\mathrm{med}}$ is the median value for the 22 wheat CGM models that use a constant *K* value (Table 2). Simulations were performed using the wheat CGM *SiriusQuality*. Data are mean ± 1 sd for 30 growing seasons by 5 cultivars.

To quantify the uncertainty in crop growth and grain yield simulations due to light extinction models, we implemented the FIPAR models we analyzed in this study in the CGM *SiriusQuality*, and then simulated total biomass production and yield at five sites with contrasted illumination conditions. The $K_{\mathrm{cst}}^{}$ model underestimated by 16% (in Wad Medani, The Sudan) to 50% (in Jokiolinen, Finland) the cumulative IPAR at maturity compared with the $K_{\mathrm{ell}}^{C}\;$model (Figure 7A). The $K_{\mathrm{cst}}^{\mathrm{HS}}\;$model performs similarly to $K_{\mathrm{cst}}^{}$ model, with a slight improvement. As expected, the differences were higher at high latitudes where on average the solar elevation angle is lower and f is higher. The other four models ($K_{\mathrm{avr}}^{}$, $K_{\mathrm{sph}}^{}$, $K_{\mathrm{sph}}^{C}$, and $K_{\mathrm{ell}}^{}$) overestimated the cumulative FIPAR at maturity for all the sites compared with the $K_{\mathrm{ell}}^{C}\;$model. The $K_{\mathrm{sph}}^{C}$ model was the closest to the $K_{\mathrm{ell}}^{C}$ model; on average it overestimated cumulative IPAR by 5%. The relative differences in simulated total above ground biomass (Figure 7B) and grain yield (Figure 7C) between the $K_{\mathrm{ell}}^{C}$ model and the other models were similar to those for the cumulative IPAR. On average, simulated grain yield was 26% and 17% lower for the $K_{\mathrm{cst}}^{}$ and $K_{\mathrm{cst}}^{\mathrm{HS}}$ models compared with the $K_{\mathrm{ell}}^{C}$ model, while it was 17%, 17%, 6%, and 11% higher for the $K_{\mathrm{avr}}^{}$, $K_{\mathrm{sph}}^{}$, $K_{\mathrm{sph}}^{C}$, and $K_{\mathrm{ell}}^{}$ models compared with the $K_{\mathrm{ell}}^{C}$ model (Figure 7C).

Some CGMs that use the $K_{\mathrm{cst}}^{}$ model and the LUE approach (Monteith, 1977) to simulate biomass production increase LUE on cloudy days to account for the greater contribution to biomass accumulation by the leaves that are shaded under clear sky conditions. We, therefore, performed simulations with *SiriusQuality* that considered the increase of LUE on days with high f value for the $K_{\mathrm{cst}}^{}$ and $K_{\mathrm{cst}}^{\mathrm{HS}}$ models that do not separate the diffuse and direct components of the incoming PAR (Supplemental Figure S4). Results showed that the relative differences in grain yield between the $K_{\mathrm{cst}}^{}$ or $K_{\mathrm{cst}}^{\mathrm{HS}}$ model and the $K_{\mathrm{ell}}^{C}$ model were halved for Jokiolinen when considering the increase of LUE as f increased. In Schleswig and Vantas Huelma, the relative difference in grain yield was then smaller than 5%. In Wad Medani, the $K_{\mathrm{cst}}^{}$ and $K_{\mathrm{cst}}^{\mathrm{HS}}$ models overestimated grain yield compared with the $K_{\mathrm{ell}}^{C}$ model when the response of LUE to f was considered.

## Discussion

The review of measured *K* values reported in the literature showed a large variability. Measurements were achieved under specific illumination conditions that varied among studies. Further, the inconsistency of area index, including or not including the stems and ears, and which was used to derive *K* from Equation 1, makes the reported *K* values difficult to compare. Considering the diversity of growth conditions and cultivars used in the studies reported in the literature, no clear conclusions could be drawn to explain the wide range of variability of *K* and the possible drivers. The lack of common protocol for field measurements of *K* also contributes to the diversity of values reported. A more physically based approach is necessary to better understand the limitations associated with these reported *K* values estimated from field measurements.

The compilation of light interception models used in wheat CGMs shows that most of them use a simplified formulation with a constant *K*. The median value of *K* = 0.52 used in wheat CGM is close to the median value calculated from measurements of FIPAR and GAI in the field (*K* = 0.59). However, very few models provide justifications for these values. Further, the use of $K_{\mathrm{cst}}^{}$ does not account for the illumination conditions experienced by canopies or differences in canopy structure observed between growth stages and cultivars. Some empirical models were proposed to drive *K* values depending on growth stages (Brown et al., 2018a, 2018b) or sowing patterns (Dzotsi et al., 2013). The two other FIPAR models used in CGMs are physically based, assuming the distribution of canopy element inclination is equal to a single average value ($K_{\mathrm{avr}}^{}$) or follows a spherical distribution accounting ($K_{\mathrm{sph}}^{C}$) or not ($K_{\mathrm{sph}}^{}$) for leaf clumping. However, these three models are limited by their lack of flexibility as well as realism when compared to reference FIPAR values.

Therefore, we proposed a physically based model ($K_{\mathrm{sph}}^{C}$) assuming that the surface inclination distribution follows an ellipsoidal distribution with some possible leaf clumping. We demonstrated that the distribution of surface inclination of green elements in wheat canopies is well represented by an ellipsoidal distribution with an average inclination angle that depends on the growth stage and cultivars. Further sensitivity analysis using the $K_{\mathrm{sph}}^{C}$ model illustrated the importance of taking into account the illumination conditions, particularly for the vegetative growth stages with low to medium GAI values, when ${\mathrm{FIPAR}}_{\mathrm{day}}^{}$ is very sensitive to the illumination conditions.

A clumping factor was required to better describe FIPAR as a function of GAI. It varies mostly with row spacing, with a value close to 0.79 for 17.5 cm row spacing and 0.67 for 35 cm row spacing. These values are lower than the value of 0.9 previously reported for single-row spacing wheat canopies (Baret et al., 2010). This difference may be partly explained by the error in GAI measurements, which is usually ˃10%. The 3D reconstruction of the canopies did not account for the twisting of leaves, which may also contribute to the difference in $C_{\mathrm{GAI}}$ between our study and previous ones. It should also be noted that $C_{\mathrm{GAI}}$ was assumed here to be independent from the row and solar directions, while the clumping factor may vary by almost 30% as reported by (Peng et al., 2018).

We showed that the ellipsoidal model outperformed the other models for most illumination conditions, but the differences between the models decreased for medium to high GAI values because of the saturation of FIPAR as a function of GAI. This explains why at GS39 the differences in cumulative IPAR between models were only 2–10% when all models used the same GAI trajectory. This conclusion is supported by several sensitivity analyses of CGMs that use the $K_{\mathrm{cst}}^{}$ model, which shows that variations of *K* of 20% around it is nominal value (i.e. about two-third of uncertainty of *K* reported for wheat CGMs) alone explain <5% of the yield variance (Makowski et al., 2006; Martre et al., 2015a, 2015b, 2015c; Casadebaig et al., 2016). However, small differences in FIPAR during the early growth stages may have large effects on crop growth and final biomass and yield (Asseng et al., 2003; Bassu et al., 2011; Wilson et al., 2015). Our simulations with the wheat CGM *SiriusQuality* confirmed that, integrated over the growing season, the differences in FIPAR simulated by the different light interception models used in wheat CGMs may cause large uncertainty in simulated wheat growth and final grain yield. We showed that considering the response of LUE to f in models that use a constant *K* and the LUE approach may partly compensate for the underestimation of light interception by the canopy, in particular at high latitudes where the solar elevation angle is lower or when f is high.

Our results are supported by detailed 3D canopy architecture reconstructions achieved under specific conditions. We considered contrasting cultivars, growth stages, and sowing density that represent a large variability of wheat canopy structure. However, we focused on the growth stages between the beginning of stem extension and flag leaf ligule. Similar efforts should therefore be taken to describe the periods after heading. In wheat canopies, ears intercept 10–30% of the incident PAR at the top of the canopy (Thorne et al., 1988; Bertheloot et al., 2008). Future work should also consider the post-flowering period where GAI decreases because of leaf senescence, when the ear layer may contribute even more to intercept PAR.

One main advantage of using a realistic physically based FIPAR model is to provide more accurate values of radiation use efficiency (RUE). When LUE is calibrated from biomass measurements and estimates of IPAR using a too simple model, errors in FIPAR computation will induce biases in LUE estimated values, particularly for the early growth stages. The proposed physically based light interception model may reduce uncertainties in yield prediction from CGMs based on calibrated RUE values (Sinclair and Muchow, 1999).

The engineering of photosynthetic process has been considered as promising to increase the genetic yield potential of crops (Long et al., 2015; Wu et al., 2019). The use of a realistic FIPAR model appears thus very important when exploiting RUE or leaf photosynthesis traits as functional traits that characterize genotypes for plant breeding. This requires the light interception model to be established physically on realistic representations of the canopy, and also its interaction with the incident radiation. The proposed ellipsoidal model offers the possibility to compute the fraction of sun lit and shaded green elements in canopies (Pury and Farquhar, 1997) and to consider several layers in the canopy (Sellers et al., 1992; Wang and Leuning, 1998). Such a model is described and the source code is provided in Manceau et al. (2020).

The rapid progress of high-throughput phenotyping technology offers affordable and operational systems based on very high-resolution imagery. It allows measuring accurately the green fraction in several directions from which the two key parameters of the $K_{\mathrm{ell}}^{C}$ model, namely $C_{\mathrm{GAI}}^{}$ and $\bar{\theta }$, can be estimated as a function of the genotype and growth stage (Liu et al., 2019). This would be of great interest for breeders to design ideotypes better adapted to given scenarios.

## Conclusion

We reviewed the light interception models used in CGM for wheat crops. The fraction of intercepted radiation is always related to the area of green elements per unit ground area through an exponential law characterized by an extinction coefficient, K. Experimental observations of K show that it ranges between 0.28 and 0.90 with a median value of 0.59. This wide variability is explained by the wide range of FIPAR measurement methods, illumination and growth conditions as well as genotype variability. Further, while GAI that accounts for all the green surfaces should be considered, some studies do not account for the stems and ears that may represent a large fraction of the green surfaces. Most of the CGMs use a constant K with a median value of K = 0.52 with, however, a large variability between CGMs. To explicitly account for the changes in illumination conditions, few models use a physically based model assuming either that the surfaces are inclined at a single angle, or that the distribution of the inclination of the green surfaces follows a spherical distribution. However, our experimental observations demonstrate that the distribution of the inclination of leaf and stem surfaces is well described by an ellipsoidal distribution characterized by $\bar{\theta }$. Further, $\bar{\theta }$ may change with cultivar, growth stages and conditions. Additionally, a clumping factor should be accounted for to describe the non-perfectly random spatial distribution of green elements in the canopy volume. We, therefore, propose this physically based model using an ellipsoidal distribution of surface inclination with a clumping factor. This light interception model was compared to the other models used in CGMs based on 3D wheat canopy scenes reconstructed from detailed field measurements. Results show that it outperforms all the other models to describe the variation of FIPAR due to illumination conditions, genotype effects due to variations of surface inclination distribution, and canopy structure effects due to variation in row spacing. These differences are enhanced for the earlier stages. We then simulated the time course of FIPAR with these several light interception models using the measured time course of GAI at Grignon, France. Results show that the differences in terms of cumulated IPAR are not very large between light interception models, with most differences appearing during the early stages. We finally evaluated the impact of the light interception model used in the *SiriusQuality* CGM on biomass production and yield. This was done over five sites spanning from 14° to 60° latitude, representing a wide range of illumination and growth conditions. Results show clearly the large influence of the light interception model, particularly for the high latitude sites with particular illumination conditions. These large differences are explained by the fact that a change in FIPAR impacts the biomass production and GAI dynamics. This expresses mostly during the early growth stages. However, these early differences impact the whole fate of the crop.

It is finally concluded that the light interception model is critical for realistic simulations in CGMs. We proposed a physically based model that represents the surface inclination distribution by an ellipsoidal distribution with a clumping factor. However, this poses the problem of describing the average surface inclination angle that characterizes the ellipsoidal distribution and the clumping factor, along with their possible variation with genotypes, stages, and environmental conditions. Further work should, therefore, be directed to the development of high-throughput methods to measure the average surface inclination angle and clumping factor to better understand and model their variability.

## Materials and methods

### Crop growth conditions

Experiments were conducted during the 2012–2013 growing season at Thiverval-Grignon, France (48°51′ N, 1°58′ E). The experimental design and growth conditions are described in detail in Abichou et al. (2019). Five winter wheat (*T. aestivum*) cultivars with contrasting leaf inclination (Apache, Caphorn, Maxwell, Renan, and Soissons) were sown on October 2, 2012 in a deep loamy soil at a density of 170 seeds m^−2^ with either standard (SS, 0.175 m) or double (DS, 0.350 m) row spacing. The mean plant spacing on the row were 2.9 and 1.4 cm for SS and DS, respectively, ensuring similar plant density. The experimental design included two blocks (15 × 1.75 m) per treatment. Crops were grown under nonlimiting water and nitrogen conditions and kept free of weeds and diseases. N fertilization followed the recommended scheme for the area, with one dose at tillering and one dose applied shortly before stem extension. Air temperature at 2 m and global and diffuse PAR were recorded by a meteorological station located next to the experimental field. Thermal time was calculated from plant emergence on an hourly basis, assuming a linear response of plant development to temperature, with a base temperature of 0°C.

### Plant digitalization and 3D canopy reconstruction

**Figure 8:**
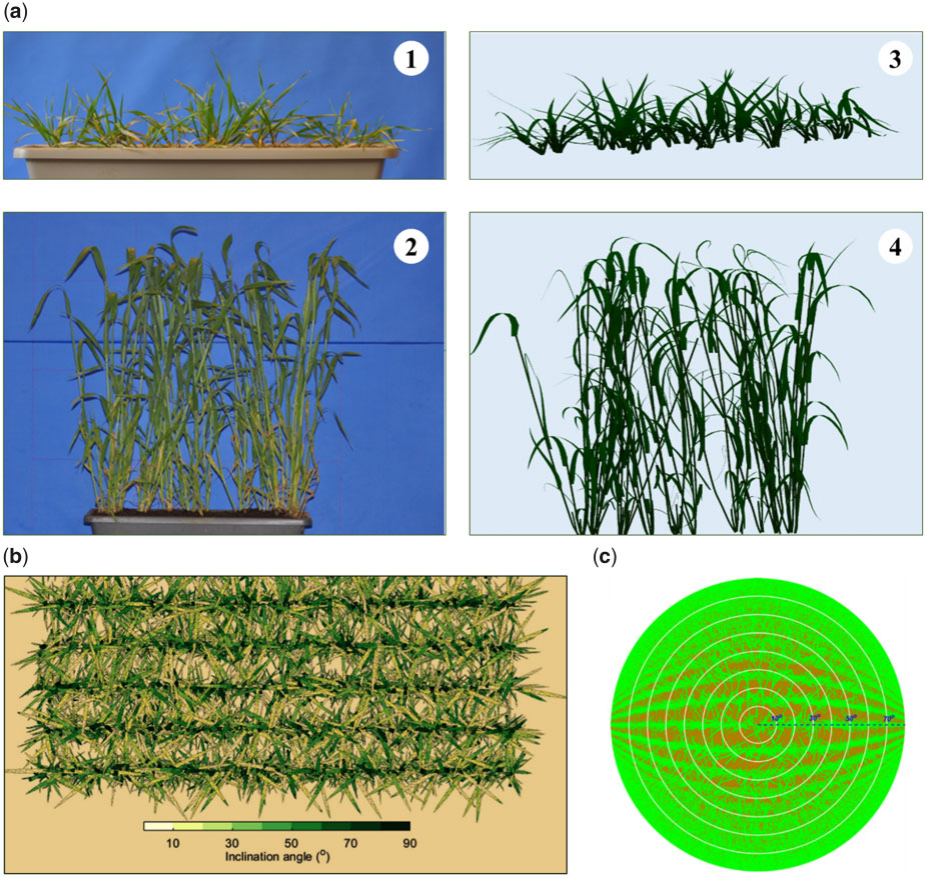
Input and output of D3P to estimate FIPAR, GAI, $C_{\mathrm{GAI}}$ and $\bar{\theta }$: (A) photo of a row of transplants from the field (a-1 and a-2) and 3D reconstructed images (a-3 and a-4) of the winter wheat cultivar Maxwell at growth stages 31 (a-1 and a-3) and 39 (a-2 and a-4). (B) Nadir view of the reconstructed 3D wheat canopy where the inclination angle of each triangle is color coded; (C) Hemispherical image with a 160^**°**^ field of view over the simulated canopy.

Canopies of each cultivar for the two row-spacing treatments were digitized at the beginning of stem elongation (GS31) (Zadoks et al., 1974) and the ligulation of the flag leaf (GS39). In each plot, two 0.4-m long segments of one row were collected, including the soil and the roots down to 15-cm depth. Row segments were put intact in plastic containers and then brought to the laboratory where the plant structure was digitized (Figure 8A). Great care was paid in this process to preserve the original canopy structure. Each row segment was photographed (Figure 8A), then the coordinates of points along leaves midribs and stems were recorded with a magnetic digitizer (3SpaceE Fastrak, Polhemus Inc., Colchester, VT, USA) using the 3A software (Adam et al., 1999). Apart from the skeleton information, the maximum width and length of the lamina of the individual mature leaves of the main stem, and the stem diameter and leaf collar height of each individual leaf were measured with a ruler on 30 tagged plants. The corresponding lamina width and shape were derived using empirical parametric models (Dornbusch et al., 2011; Abichou, 2016). The 3D canopy architecture was then reconstructed as a 3D mesh composed of triangles using the 3D plant structure modeling library PlantGL (Pradal et al., 2009).

### Calculation of GAI, average surface inclination, clumping factor, and FIPAR from 3D digitalized canopies

All the green triangles in the reconstructed 3D canopies were labeled as lamina or stem. ${\mathrm{GAI}}_{\mathrm{mes}}$ was calculated as the sum of the area of all the triangles for the leaves, and only half the sum of the area of the stem triangles (Lang, 1991; Chen and Black, 1992). ${\mathrm{GAI}}_{\mathrm{eff}}$ was calculated as (Chen and Black, 1991; Weiss et al., 2004):

(25) $${\mathrm{GAI}}_{\mathrm{eff}}=2\int_{0}^{\pi /2}-\ln(1-\mathrm{GF}(\beta ))\cos\;\beta \;\cos\;\beta \;d\beta$$

$C_{\mathrm{GAI}}$ was then calculated as the ratio of ${\mathrm{GAI}}_{\mathrm{mes}}$ to ${\mathrm{GAI}}_{\mathrm{eff}}$ (Eq. 18). $\bar{\theta }$ was calculated as the average of inclination angles of all lamina and stem triangles weighted by their corresponding areas (Figure 8B).

${\mathrm{FIPAR}}^{\mathrm{dir}}$ and ${\mathrm{FIPAR}}^{\mathrm{dif}}$ were calculated from the 3D reconstructed canopies using the Digital Plant Phenotyping Platform (D3P; Liu et al., 2019). The virtual 3D plants were duplicated to create 5 × 5 m scenes with row spacing consistent with the experiment. Then the RGB camera simulator in D3P was used to render hemispherical photos over the virtual scenes. Photographs were taken 30 cm above the top of the reconstructed 3D canopies with a 160^°^ field of view and a 25 million pixel resolution. The camera was moved 7 times in a diagonal pattern between the two central rows to account for the row effect. The camera was maintained horizontal and parallel to the row direction. Thus, the directional green fraction was symmetric along and across the row direction. No azimuthal effect was considered, and images were processed by rings of 10° elevation angle ($\beta_{i}-5{}^{\circ}<\beta <\beta_{i}+5{}^{\circ}$) to compute ${\mathrm{FIPAR}}^{\mathrm{dir}}$ (Figure 8C). ${\mathrm{FIPAR}}^{\mathrm{dif}}$ was then calculated by using Equation 24. These ${\mathrm{FIPAR}}^{\mathrm{dir}}$ and ${\mathrm{FIPAR}}^{\mathrm{dif}}$ values were used as references to evaluate the performance of the FIPAR models reviewed above.

### Calculation of FIPAR dynamics

The different FIPAR models were first intercompared at Grignon, France. Calculations were done using $\bar{\theta }$ and ${\mathrm{GAI}}_{\mathrm{mes}}$ values for the cultivars Maxwell and Caphorn and the single row-spacing treatment at Grignon.$\;{\mathrm{GAI}}_{\mathrm{mes}}$ was measured weekly from the ligulation of the fourth leaf to GS39 from down-looking RGB photographs (Supplemental Methods S2). The measured ${\mathrm{GAI}}_{\mathrm{mes}}$ values were interpolated linearly as a function of thermal time. For the ellipsoidal models, we assumed that $\bar{\theta }$ was constant between crop emergence and GS31 and then increased linearly between GS31 and GS39. These assumptions are consistent with the ontogenic changes in lamina insertion angles reported by Abichou et al. (2019). We further assumed that the clumping factor did not change between crop emergence and GS39. Calculations were done for the 2012–2013 growing season at Grignon, France. The hourly PAR intercepted by canopies (IPAR) was calculated as the incident PAR times FIPAR. The daily IPAR was then calculated as the sum of hourly IPAR, assuming $f_{\mathrm{PAR}}^{\mathrm{dif}}$ was constant hourly.

### Evaluation of the impact of light extinction coefficient models on simulated total above-ground biomass and grain yield

To quantify the impact of light extinction coefficient models on simulated wheat crop growth and grain yield uncertainty, we created a model component in the BioMA software framework that implements the light extinction coefficient and FIPAR models analyzed in this study (Manceau et al., 2020) and integrated it in the wheat CGM *SiriusQuality* (Martre et al., 2006). In *SiriusQuality*, biomass production is modeled using the LUE approach (Monteith, 1977). LUE (and biomass production) is calculated for each leaf cohort (Martre and Dambreville, 2018) and is modified by the specific leaf N mass of the leaf cohort (Sinclair and Horie, 1989), canopy temperature (Wang et al., 2017), soil water deficit (Jamieson et al., 1998), atmospheric CO_2_ concentration (Jamieson et al., 2000), and the fraction of diffuse radiation. However, LUE was assumed as constant, except for special simulations to assess whether the LUE response to the fraction of diffuse radiation considered in some CGMs that use a constant *K* and the LUE approach to model biomass production can account for the higher FIPAR by the canopy on a cloudy day (see Supplemental Methods S3).

*SiriusQuality* was executed for a large range of illumination conditions at five sites spanning the latitudes at which wheat is grown (Supplemental Table S4): Wad Medani, The Sudan (14° 24′ N), Ventas Huelma, Spain (37° 9′ N), Grignon, France (48°51′ N), Schleswig, Germany (54° 31′ N), and Jokioinen, Finland (60° 48′ N). At all sites but Grignon, simulations were set up using the observed local mean sowing dates, initial soil N and water contents, crop management, daily weather data, soil characteristics, and cultivars reported by Asseng et al. (2019). In Grignon, simulations were set up using similar information provided in (Jeuffroy and Bouchard, 1999) and (Jeuffroy and Recous, 1999), and daily weather data from the local INRAE weather station. The fraction of diffuse PAR (f) was calculated with the approach proposed by Spitters et al. (1986) used in *SiriusQuality*.

Simulations were done with each of the seven light extinction coefficients and FIPAR models for 30 consecutive years (1981–2010), with initial conditions reset each year and using the same parameters and inputs at each site for all the light extinction models. Cultivar parameters were previously calibrated against the observed anthesis and maturity dates by considering information on vernalization requirements and photoperiod sensitivity (Supplemental Table S4). For the ellipsoidal models, $\bar{\theta }$ values were from the five cultivars for the single row spacing treatment at Grignon. As above, we assumed that $\bar{\theta }$ was constant between crop emergence and GS31 and then increased linearly between GS31 and GS39, and that the clumping factor did not change between crop emergence and maturity. Using the $K_{\mathrm{ell}}^{C}$ model as a reference, the relative difference from the other six models was calculated for cumulative IPAR, total above ground biomass, and grain yield.

### Statistical analyses

The distributions of the tilt angle of all the triangles from the digitalized canopies were compartmented with an ellipsoidal or spherical distribution using two-sample Kolmogorov–Smirnov tests. Differences in $\bar{\theta }$ and $C_{\mathrm{GAI}}$ due to the growth stage and row spacing were analyzed using two-way analysis of variance after verifying that the treatment effects were normally distributed with equal variance.

The accuracy of ${\mathrm{FIPAR}}^{\mathrm{dir}}$ and ${\mathrm{FIPAR}}^{\mathrm{dif}}$ calculated using the different approaches reviewed above was assessed with the RMSE:

(26) $$\mathrm{RMSE}=\sqrt{\frac{1}{n}\sum_{i=1}^{n}{\left(y_{i}-\hat{y_{i}}\right)}^{2}}$$

where $y_{i}$ and $\hat{y_{i}}$ are the predicted values and the measured values, respectively.

## Code availability

All *K* and FIPAR models presented here were coded in Matlab and we also developed an independent executable component in the BioMA software framework (http://www.biomamodelling.org), which can easily be extended and coupled with CGMs. The source code and the standalone executable of the BioMA component are freely available at https://zenodo.org/record/3820386. The *SiriusQuality* model can be downloaded from https://github.com/SiriusQuality.

## Supplemental data

The following supplemental materials are available.

**Supplemental Method S1.** Sensitivity analysis of PROSAIL model.

**Supplemental Method S2.** Measurements of effective GAI.

**Supplemental Method S3.** Modeling the response of LUE to the fraction of diffuse light.

**Supplemental Results** Validity of the FIPAR approximation.

**Supplemental Figure S1.** Relationship between sampled average inclination angle of green elements in the canopy ($\bar{\theta }$) and GAI.

**Supplemental Figure S2**. Comparison between FIPAR and FAPAR for different leaf chlorophyll concentrations (Cab) and soil reflectance ($r_{sl}$).

**Supplemental Figure S3.** Distributions of inclination angle of canopies’ green elements.

**Supplemental Figure S4.** Differences between among light extinction models.

**Supplemental Table S1.** Parameters sampling to build the dataset for FIPAR and FAPAR comparison using the PROSAIL model.

**Supplemental Table S2.** Average inclination angle of green leaf and stem elements measured in the canopy at the beginning of stem extension (GS 31) and flag leaf ligule (GS 39) for five winter wheat cultivars grown in the field with standard (17.5 cm, SS) and double (35 cm, DS) row spacing.

**Supplemental Table S3.** Two-way analysis of variance (ANOVA) for average inclination angle at canopy scale at the beginning of stem extension (GS 31) and flag leaf ligule (GS 39).

**Supplemental Table S4.** Location, cultivar, and phenology information at the five sites used to simulate wheat crop growth and grain yield with the wheat crop model *SiriusQuality*.

## Supplementary Material

kiab113_Supplementary_Data

## Notes

S.L., F.B., M.W., and P.M. planned and designed the research; MA. and B.A. designed and performed the field experiments; L.M. wrote the BioMA model component and performed the simulations; S.L. analyzed the data and wrote the first draft; P.M. supervised and completed the writing with contributions of all the authors.

The author responsible for distribution of materials integral to the findings presented in this article in accordance with the policy described in the Instructions for Authors (https://academic.oup.com/plphys/pages/general-instructions) is: Pierre Martre (pierre.martre@inrae.fr).
