# Supplementary material for: Importance of the description of light interception in crop growth models
Source: Plant Physiol. 2021 Mar 12;186(2):977–97. doi: 10.1093/plphys/kiab113 (PMC8253170; doi:10.1093/plphys/kiab113)
Supplement: kiab113_Supplementary_Data [file kiab113_supplementary_data.pdf]

Supplemental Information

Importance of the description of light interception in crop growth models

Shouyang Liu, Frédéric Baret, Mariem Abichou, Loïc Manceau, Bruno Andrieu, Marie Weiss and Pierre Martre

SUPPLEMENTAL METHODS

S1. Sensitivity analysis of PROSAIL model

In order to evaluate our hypothesis that FIPAR is an accurate proxy of FAPAR, we used PROSAIL model (Jacquemoud et al., 2009) to calculate reference FAPAR for a wide range of canopy structures, soil and leaf optical properties and solar configurations (Table S1). PROSAIL model is popular to simulate radiative transfer in the canopy, which allows taking into account variations of leaf biochemical content and soil optical property. The corresponding FIPAR was computed using the model proposed in this work. Note that to account for the covariance of GAI and $\bar{\theta}$, the sampling was conducted with prior knowledge from the field experiments carried at Grignon (Fig. S1). The sensitivity analysis of PROSAIL to soil reflectance and leaf optical properties was carried out for fifty combinations of GAI and $\bar{\theta}$, three values of f corresponding to clear sky conditions, overcast conditions, and an intermediate condition, and three values of β spanning the range of sun elevation angle (Table S1). Regarding leaf optical property, chlorophyll concentration is the dominant factor (Jacquemoud and Baret, 1990). PROSAIL does not allow accounting for the impact of spatial variations of optical properties within the canopy. The range of chlorophyll concentration was thus defined based on values reported for wheat canopies grown in the field (Jiang, 2019; Li et al., 2020), instead of that at leaf scale. This partly overcomes the limitation of PROSAIL model and increases the reliability of our assessment for the FIPAR approximation. For soil reflectance, the minimum value was set at 0.01 (that is close to black soil), while the maximum was set at 0.3 (Jacquemoud et al., 1992).

S2. Measurements of effective GAI

GAI_{eff} of each cultivar and sowing density was measured weekly from the ligulation of the fourth leaf to GS39. At each measurement date and in each plot, we took six down looking photographs at 57° zenithal angle with a Nikon D90 digital camera with a focal length of 50 mm which defines a field of view of 26.6° × 18°. Photographs were taken 2.2 m above the ground level and covered an area of 2.03 m^2. Photographs were processed using the CAN-EYE software (https://www6.paca.inrae.fr/can-eye) to determine the proportion of vegetation pixels, which corresponds to the green fraction (GF). GAI_{eff} was then calculated as (Baret et al., 2010):

$$\mathrm{GAI_{eff}} = -\frac{\cos 57.5^{\circ}}{0.5}\log\left(\mathrm{GF}(57^{\circ})\right) \quad \text{(S1)}$$

S3. Modeling the response of LUE to the fraction of diffuse light

Some CGMs that use a constant light extinction coefficient and the LUE approach to model biomass production, consider the increase of LUE as f increases or PAR decreases (Table 2). Corrections of LUE to illumination conditions have been introduced in these models to account for the higher LUE on cloudy day due to the greater contribution to biomass accumulation of shaded leaves, with high photosynthesis efficiency, in the lower layers of the canopy (Sinclair et al., 1992; Hammer and Wright, 1994; Rodriguez and Sadras, 2007). Therefore, we also performed simulations with *SiriusQuality* by considering, for the models that do not separate the diffuse and direct PAR compounds (i.e. K_{cst}, and $K_{\mathrm{cst}}^{\mathrm{HS}}$), the increase of LUE under diffuse light conditions. For these simulations, LUE was calculated as:

$$\mathrm{LUE}(i) = \begin{cases} \mathrm{LUE_{dir}}, & f(i) < 0.23 \\ \mathrm{LUE_{dir}} + s_{\mathrm{FDR}}(f(i) - 0.23), & f(i) \geq 0.23 \end{cases} \quad \text{(S2)}$$

where s_{FR} (g DM MJ^{-1} PAR) is the slope of the relationship between LUE and f, $\mathrm{LUE_{dir}}$ (g DM MJ^{-1} PAR) is the LUE for direct light, and $f(i)$ is the fraction of diffuse radiation of the PAR incident on the ith leaf layer counted from the base of the canopy. $\mathrm{LUE_{dir}}$ was set equal to 2.27 g DM MJ^{-1} PAR (Sinclair and Muchow, 1999) and s_{FDR} to 1.6 g DM MJ^{-1} PAR (Rodriguez and Sadras, 2007). Assuming that the diffusion of light within the canopy is sufficiently similar to that in the atmosphere, $f(i)$ was calculated using the equation derived by Spitters et al. (1986) given by:

$$f(i) = \begin{cases} 1, & S(i) < 0.07 \\ 1 - 2.3 \times (S(i) - 0.07)^2, & 0.07 \leq S(i) < 0.35 \\ 1.33 - 1.46 \times S(i), & 0.35 \leq S(i) < 0.75 \\ 0.23, & S(i) \geq 0.75 \end{cases} \quad \text{(S3)}$$

where $S(i)$ (dimensionless) is the canopy transmission ratio at the top of leaf layer *i* given by:

$$S(i) = S\frac{\mathrm{PAR_i}(i)}{\mathrm{PAR_0}} \quad \text{(S4)}$$

where S (dimensionless) is the atmospheric transmission ratio, that is the ratio of global solar radiation to the extra-terrestrial radiation; and $\mathrm{PAR_i}(i)$ (MJ^{1} PAR m^{-2} (ground)) is the PAR intercepted by leaf lamina of leaf layer *i* given by:

$$\mathrm{PAR_i}(i) = 0.48 \times R_{\mathrm{g}} \times e^{-K\times\sum_{j=i+1}^{n}\mathrm{GAI}(j)}\left(1 - e^{-K\times A_{\mathrm{L}}(i)}\right) \quad \text{(S5)}$$

where R_{g} (MJ m^{-2} (ground)) is the global solar radiation incident at the top of the canopy, $\sum_{j=i+1}^{n}\mathrm{GAI}(j)$ (m^{2} (leaf) m^{-2} (ground)) is the cumulative GAI above leaf layer *i*; and $A_{\mathrm{L}}(i)$ (m^{2} (leaf) m^{-2} (ground)) is the leaf lamina area index of leaf layer *i*.

SUPPLEMENTAL RESULTS

Validity of the FIPAR approximation

The use of FIPAR as a proxy of FAPAR is based on the assumptions that the contribution of leaf reflectance and transmittance and soil reflectance are negligible in the budget of canopy light absorption. To evaluate the validity of these assumptions, it is necessary to compare the difference between FIPAR and FAPAR under different combinations of leaf and soil optical properties, canopy structure, illumination conditions. Therefore, we conducted a sensitivity analysis using PROSAIL model. Figure S2 shows that over a wide range of conditions, FIPAR was very close to FAPAR with generally slight underestimation. The differences between FIPAR and FAPR decreased with the increase of chlorophyll concentration, indicating low transmittance and reflectance, as well as with the decrease of the soil reflectance. Among the nine situations investigated, the situation with low chlorophyll concentration and high soil reflectance deviated the most from our black soil and leaf assumption (Fig. S2g). Even so, the difference was still acceptable with an RMSE < 0.05. The largest differences between FIPAR and FAPAR were observed for values close to 0.5 and for lower values corresponding to early growth stages. At early growth stages, more light penetrates through canopies and finally reach the soil surface. However, at early growth stage the light reflected by the soil surface is also more likely to pass through the canopy.

SUPPLEMENTAL TABLES

Supplemental Table S1 Parameters sampling to build the dataset for FIPAR and FAPAR comparison using the PROSAIL model. Definition of the parameters refers to Table 3. GAI, green area index, $\bar{\theta}$, Average surface inclination angle; r_{sl}, soil reflectance; f, Fraction of diffuse PAR to the total incident PAR; Cab, leaf chlorophyll concentration; β, Sun elevation angle.

**Parameters**	**Sampling**		**Note**	**Unit**
	Range	Number		
GAI	[0.2, 7]	100 combinations	Sampled in log distribution	m^2 (green organs) m^{-2} (ground)
$\bar{\theta}$	[38, 74]		Sampled in a uniform distribution	°
r_{sl}	[0.01, 0.3]	3	High: 0.3, Middle: 0.15, Low: 0.01	scalar
f	[0.2, 1.0]	3	High: 1.0, Middle: 0.0.6, Low: 0.2	scalar
Cab	[30, 70]	3	High: 70, Middle: 50, Low: 30	μg cm^{-2} (leaf)
β	[20, 80]	3	High: 80, Middle: 60, Low: 20	°

Supplemental Table S2. Average inclination angle of green leaf and stem elements measured in the canopy at the beginning of stem extension (GS 31) and flag leaf ligule (GS 39) for five winter wheat cultivars grown in the field with standard (17.5 cm, SS) and double (35 cm, DS) row spacing. Data are mean ± 1 sd.

Growth stage	Row spacing	Average inclination angle (°)									
		Apache		Caphorn		Maxwell		Renan		Soissons	
		Leaf	Stem	Leaf	Stem	Leaf	Stem	Leaf	Stem	Leaf	Stem
GS31	SS	41 ± 23	76 ± 12	46 ± 24	79 ± 9	30 ± 22	70 ± 14	32 ± 23	72 ± 14	36 ± 23	72 ± 13
	DS	45 ± 23	78 ± 9	45 ± 24	77 ± 11	31 ± 22	77 ± 12	42 ± 26	77 ± 11	42 ± 23	74 ± 12
GS39	SS	60 ± 21	85 ± 4	67 ± 15	85 ± 5	65 ± 17	84 ± 6	64 ± 21	84 ± 5	62 ± 18	85 ± 5
	DS	56 ± 24	84 ± 6	67 ± 14	84 ± 5	67 ± 17	85 ± 5	63 ± 21	84 ± 5	57 ± 21	84 ± 5

Supplemental Table S3. Two-way analysis of variance (ANOVA) for average inclination angle at canopy scale at the beginning of stem extension (GS 31) and flag leaf ligule (GS 39). SS: sum of squares due to each source; df: degrees of freedom associated with each source; MS: mean squares for each source, SS/df; *F*: *F*-statistic; $P > F$: probability that the *F*-statistic can take a value larger than the computed test-statistic value.

Growth stage	Source	SS	df	MS	*F*	*P* > *F*
GS 31	Row spacing	42.708	1	42.7077	6.06	0.0696
	Cultivars	194.042	4	48.5106	6.88	0.0442
	Error	28.194	4	7.0484		
	Total	264.944	9			
GS 39	Row spacing	5.1644	1	5.16441	1.37	0.3061
	Cultivars	28.1042	4	7.02604	1.87	0.2797
	Error	15.0328	4	3.75819		
	Total	48.3013	9			

Supplemental Table S4. Location, cultivar, and phenology information at the five sites used to simulate wheat crop growth and grain yield with the wheat crop model *SiriusQuality*. Data were obtained from Asseng et al. (2019) for The Sudan, Spain, Germany, and Finland, and from Jeuffroy and Recous (1999) and Jeuffroy and Bouchard (1999) for France.

				Cultivar						
**Country**	**Location**	**latitude**	**longitude**	**Name**	**Growth habit**[a]	**Cold requirement**[b]	**Photoperio d sensitivity**[b]	**Sowing date**	**Mean 50%-anthesis date**	**Mean maturity date**
The Sudan	Wad Medani	14.40	33.50	Debeira	Spring	3	2	20 Nov.	25 Jan.	25 Feb.
Spain	Ventas Huelma	37.16	-3.83	Basri Bey	Spring	4	4	18 Dec.	12 May	15 Jun.
France	Grignon	48.85	1.92	Soissons	Winter	3	3	15 Oct.	15 May	1 Jul.
Germany	Schleswig	54.53	9.55	Dekan	Winter	5	2	25 Sep.	15 Jun.	25 Jul.
Finland	Jokioinen	60.8	23.48	Steklov-24	Spring	2	2	6 Jan.	7 Jul.	15 Aug.

[a] S, spring type; W, winter type

[b] Vernalization requirement and photoperiod sensitivity of the cultivars range from nil (0) to very high (6)

SUPPLEMENTAL FIGURES

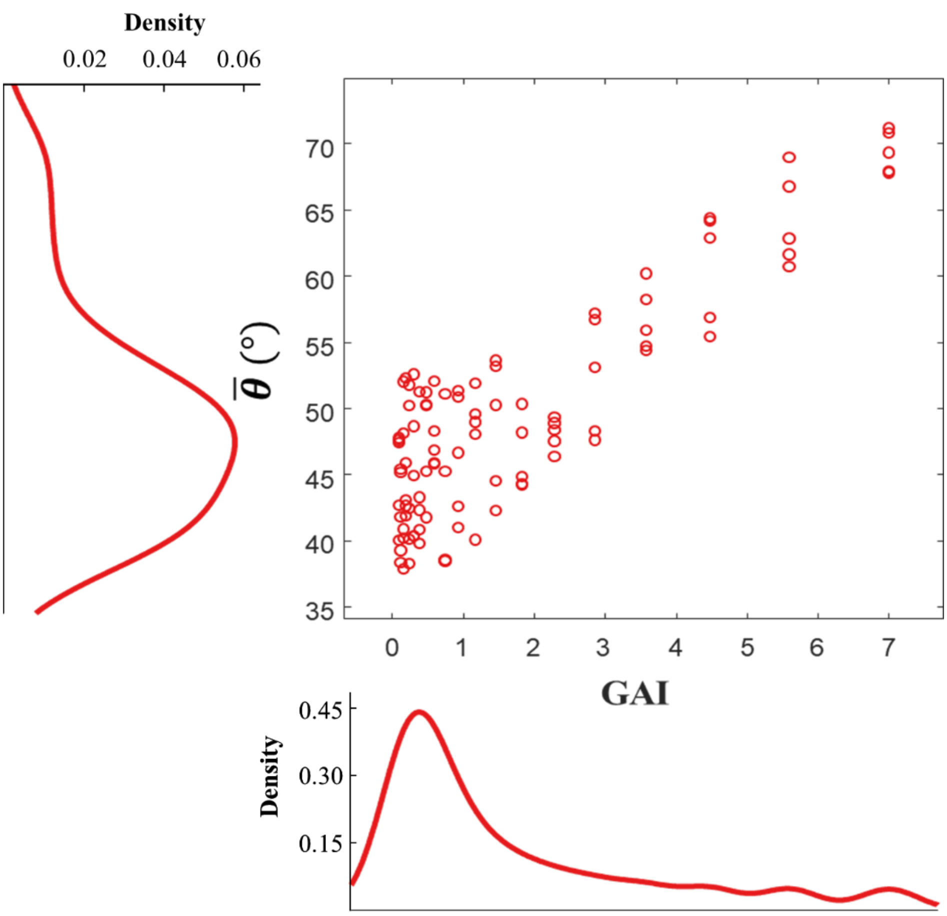

Supplemental Figure S1. Relationship between sampled average inclination angle of green elements in the canopy ($\bar{\theta}$) and green area index (GAI). Curves on the side of the axes show the distribution of $\bar{\theta}$ and GAI samples. Twenty values of GAI were sampled assuming a log distribution between 0 and 7 m^2 m^{-2}. $\bar{\theta}$ was assumed to be constant between emergence and the start of stem elongation and then increases linearly until flag ligulation. The range of $\bar{\theta}$ was determined according to the field measurement. For each sampled value of GAI, five values of $\bar{\theta}$ were sampled assuming a uniform distribution between its upper and lower bounds.

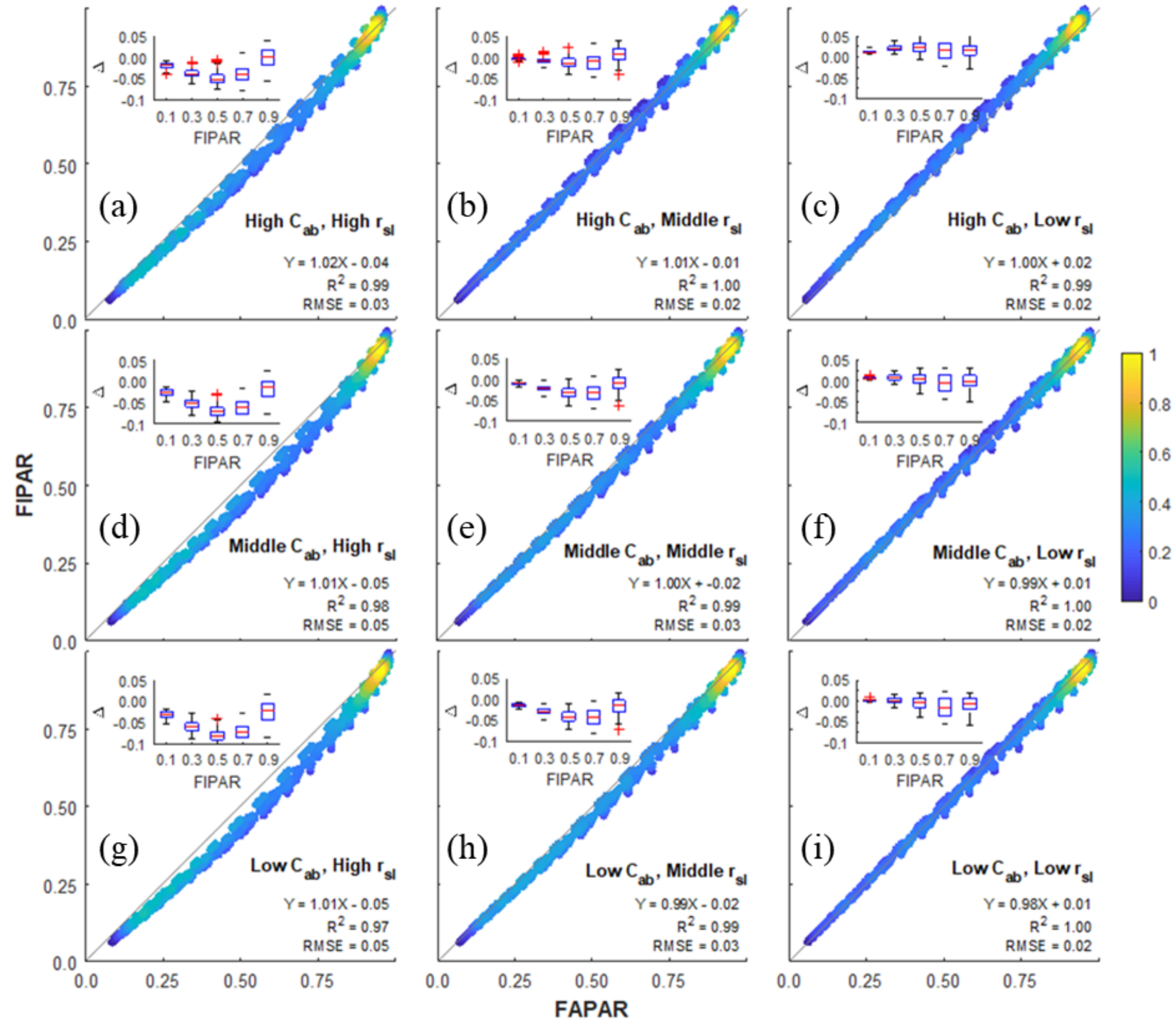

Supplemental Figure S2. Comparison between fraction of intercepted photosynthetically active radiation (FIPAR) and fraction of absorbed photosynthetically active radiation (FAPAR) for different leaf chlorophyll concentrations (Cab) and soil reflectance (r_{sl}). Simulations were done with the PROSAIL model using nine combinations of low, medium, and high Cab (30, 50, 70 µg cm^{-2}) and r_{sl} (0.01, 0.15 and 0.3). Data are for 900 samples created by varying GAI, $\bar{\theta}$, f and β (Table S1). Symbol colors indicates the relative data density. Solid grey lines are 1:1 lines. Insets show the residuals of the FIPAR versus FAPAR relationship (Δ) for different classes of FIPAR values.

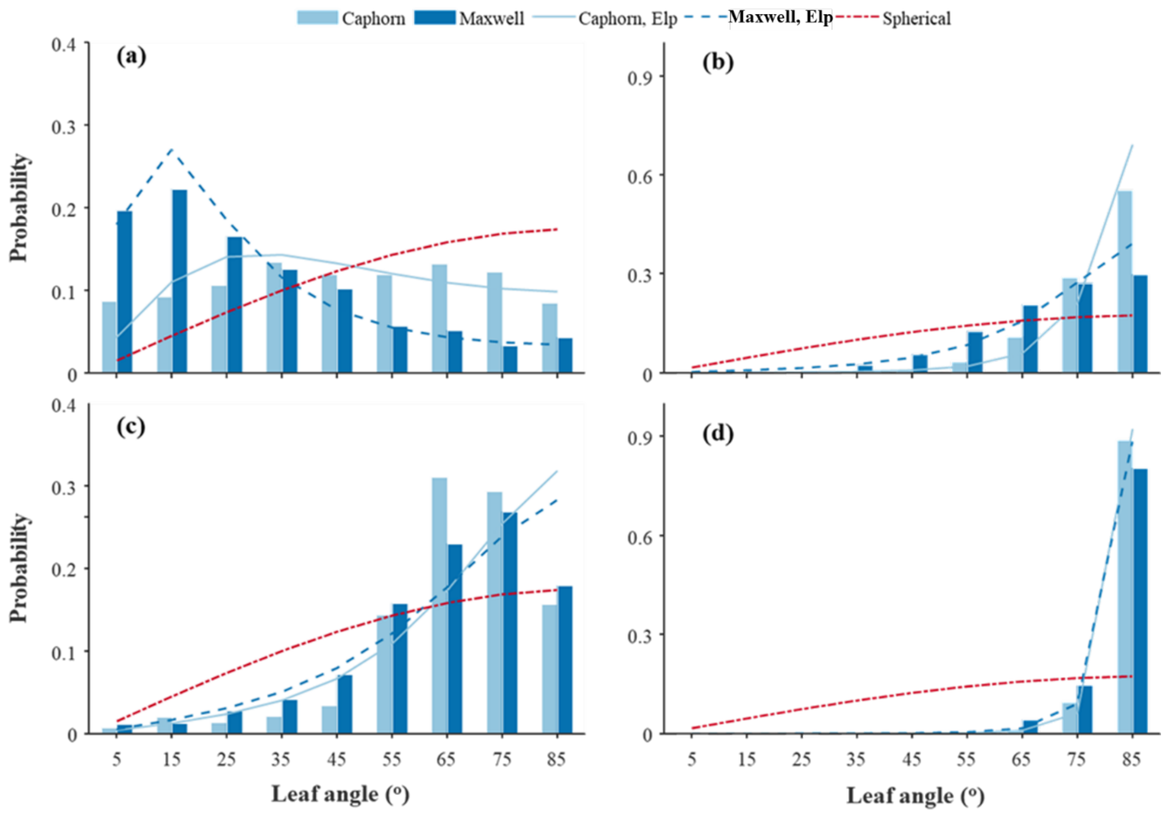

Supplemental Figure S3. Distribution of canopy surfaces' inclination angle. Leaf lamina (a and c) and stem/sheath (b and d) Inclination angle distribution at the beginning of stem elongation (GS31) and flag leaf ligulation (GS39) for the winter wheat cultivars Maxwell (dark blue) and Caphorn grown (light blue) in the field with standard row spacing. Bars are the observations extracted from 3D reconstructed canopies. Solid and dashed blue lines are the distribution calculated with the ellipsoidal model for Caphorn and Maxwell, respectively. Dot-dashed red lines are the distribution calculated with the spherical model.

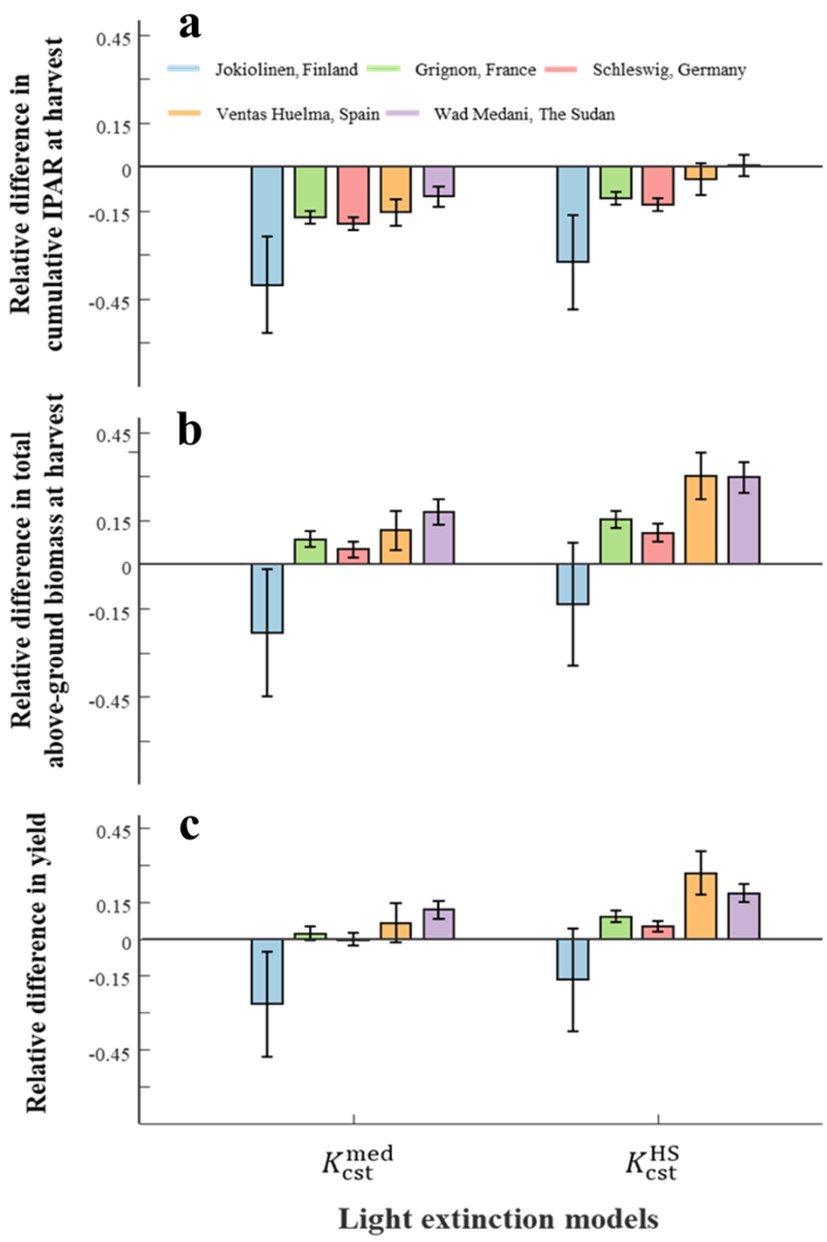

Supplemental Figure S4. Differences between different light extinction models. Relative differences in simulated cumulative intercepted PAR at harvest (a), above-ground biomass at harvest (b) and the final grain yield (c) for the $K_{\mathrm{cst}}^{\mathrm{med}}$ and $K_{\mathrm{cst}}^{\mathrm{HS}}$ light extinction models relative to the model with ellipsoidal leaf inclination angle distribution accounting for clumping ($K_{\mathrm{ell}}^{\mathrm{C}}$). Simulations were performed with the wheat crop growth model *SiriusQuality* considering the response of LUE to f (Eq. S2). For the $K_{\mathrm{cst}}^{\mathrm{med}}$ model K was set at the median value for the 22 crop growth models that use a constant K (Table 2). Simulations were conducted for 30 years (1981-2010) at five locations representing the range of latitude at which wheat is grown. Data are mean ± 1 s.d. for 30 growing seasons by 5 cultivars.

Reference

Asseng S, Martre P, Maiorano A, Rötter RP, O'Leary GJ, Fitzgerald GJ, Girousse C, Motzo R, Giunta F, Babar MA, Reynolds MP, Kheir AMS, Thorburn PJ, Waha K, Ruane AC, Aggarwal PK, Ahmed M, Balkovič J, Basso B, Biernath C, Bindi M, Cammarano D, Challinor AJ, De Sanctis G, Dumont B, Eyshi Rezaei E, Fereres E, Ferrise R, Garcia-Vila M, Gayler S, Gao Y, Horan H, Hoogenboom G, Izaurralde RC, Jabloun M, Jones CD, Kassie BT, Kersebaum K-C, Klein C, Koehler A-K, Liu B, Minoli S, Montesino San Martin M, Müller C, Naresh Kumar S, Nendel C, Olesen JE, Palosuo T, Porter JR, Priesack E, Ripoche D, Semenov MA, Stöckle C, Stratonovitch P, Streck T, Supit I, Tao F, Van der Velde M, Wallach D, Wang E, Webber H, Wolf J, Xiao L, Zhang Z, Zhao Z, Zhu Y, Ewert F (2019) Climate change impact and adaptation for wheat protein. Glob Chang Biol **25:** 155-173

Baret F, de Solan B, Lopez-Lozano R, Ma K, Weiss M (2010) GAI estimates of row crops from downward looking digital photos taken perpendicular to rows at 57.5° zenith angle: Theoretical considerations based on 3D architecture models and application to wheat crops. Agric For Meteorol **150:** 1393-1401

Hammer GL, Wright GC (1994) A theoretical analysis of nitrogen and radiation effects on radiation use efficiency in peanut. Aust J Agric Res **45:** 575-589

Jacquemoud S, Baret F (1990) PROSPECT: A model of leaf optical properties spectra. Remote sensing of environment **34:** 75-91

Jacquemoud S, Baret F, Hanocq JF (1992) Modeling Spectral and Bidirectional Soil Reflectance. Remote Sensing of Environment **41:** 123-132

Jacquemoud S, Verhoef W, Baret F, Bacour C, Zarco-Tejada PJ, Asner GP, François C, Ustin SL (2009) PROSPECT+SAIL models: A review of use for vegetation characterization. Remote Sensing of Environment **113:** S56-S66

Jeuffroy MH, Bouchard C (1999) Intensity and duration of nitrogen deficiency on wheat grain number. Crop Sci **39:** 1385-1393

Jeuffroy MH, Recous S (1999) Azodyn: A simple model simulating the date of nitrogen deficiency for decision support in wheat fertilization. Eur J Agron **10:** 129-144

Jiang J (2019) Retrieving leaf and canopy characteristics from their radiative properties using physically based models: from laboratory to satellite observations. Avignon University

Li D, Chen JM, Zhang X, Yan Y, Zhu J, Zheng H, Zhou K, Yao X, Tian Y, Zhu Y, Cheng T, Cao W (2020) Improved estimation of leaf chlorophyll content of row crops from canopy reflectance spectra through minimizing canopy structural effects and optimizing off-noon observation time. Remote Sensing of Environment **248**

Rodriguez D, Sadras VO (2007) The limit to wheat water-use efficiency in eastern Australia. I.* Gradients in the radiation environment and atmospheric demand. Aust J Agric Res **58:** 287-302

Sinclair TR, Muchow RC (1999) Radiation use efficiency. Adv Agron **65:** 216-265

Sinclair TR, Shiraiwa T, Hammer GL (1992) Variation in crop radiation-use efficiency with increased diffuse radiation. Crop Sci **32**

Spitters CJT, Toussaint HAJM, Goudriaan J (1986) Separating the diffuse and direct component of global radiation and its implications for modeling canopy photosynthesis. Part I. Components of incoming radiation. Agric For Meteorol **38:** 217-229
